# Supplementary material for: REV7 is required for processing AID initiated DNA lesions in activated B cells
Source: Nat Commun. 2020 Jun 4;11:2812. doi: 10.1038/s41467-020-16632-8 (PMC7272641; doi:10.1038/s41467-020-16632-8)
Supplement: Supplementary file 1 — Supplementary Information [file 41467_2020_16632_MOESM1_ESM.pdf]

REV7 is required for processing AID initiated DNA lesions in activated B cells

Yang et al.

Supplementary Information

Supplementary Figure 1

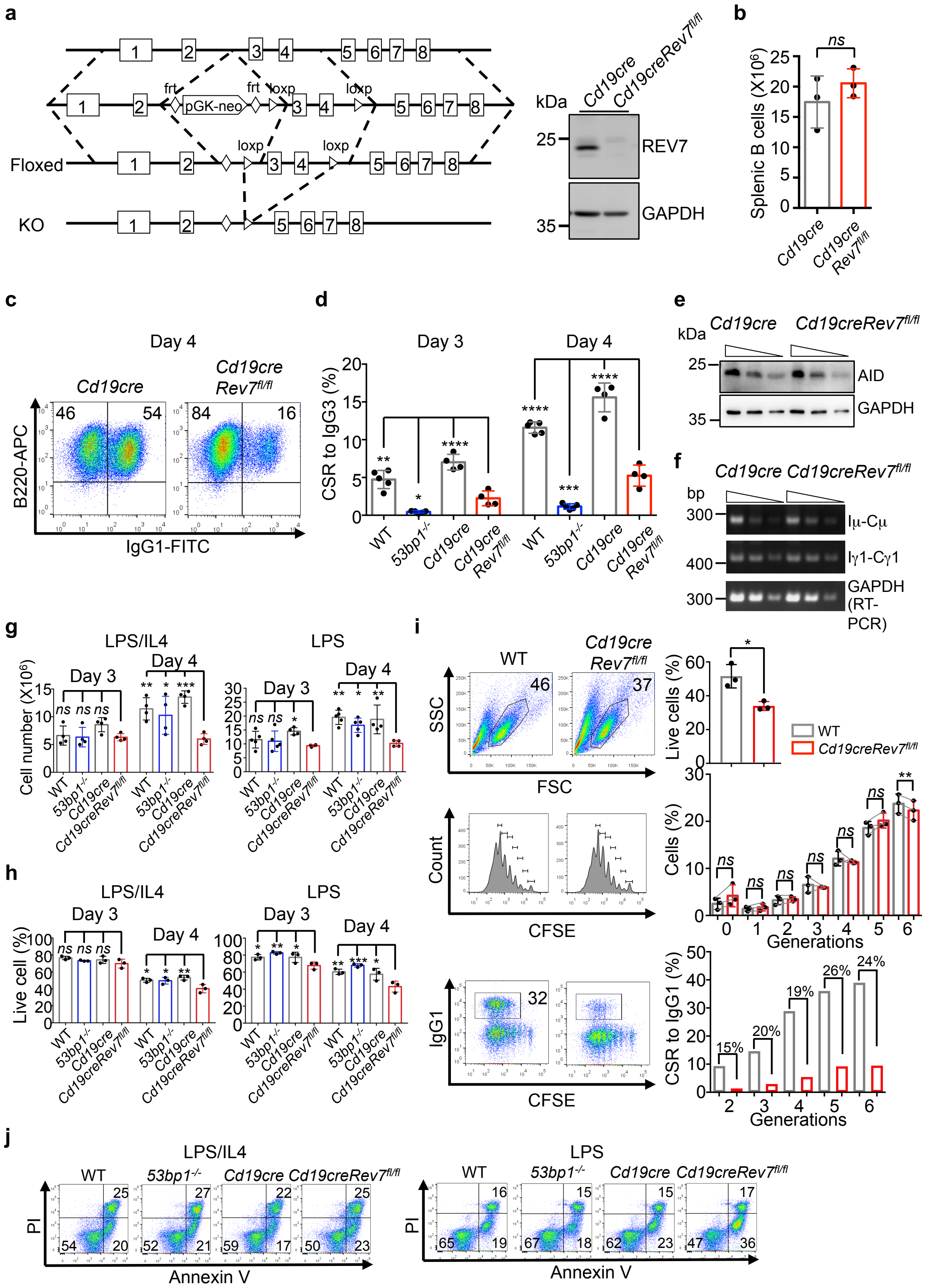

Supplementary Fig. 1. CSR in *Rev7* conditional knockout B cells.

a Illustration of *Rev7* conditional knockout strategy (left) and western blot of Rev7 protein in splenic B cells (right). Experiments were independent repeated three times, and similar results were obtained. **b** Total splenic B cell numbers of each genotype are plotted from three pairs of mice. n=3 for each genotype. **c** Representative flow cytometry blot of indicated B cells. **d** CSR levels to IgG3 at Day 3 and 4 after LPS stimulation. n=5 for WT and *53bp1*$^{-/-}$; n=4 for *Cd19cre* and *Cd19cre Rev7*$^{fl/fl}$. AID expression level (**e**) and germline transcript level (**f**) are plotted for the indicated genotypes, with two-fold serial dilutions of total cell lysate and five-fold serial dilutions of total RNA were assayed respectively. Experiments were independent repeated three times, and similar results were obtained for each repeat. Cell numbers are counted at different timepoints and plotted for LPS/IL4 and LPS stimulation (**g**). in LPS/IL4 experiment, n=4 for each genotype; in LPS experiment, n=5 for WT and *53bp1*$^{-/-}$; n=4 for *Cd19cre* and *Cd19cre Rev7*$^{fl/fl}$. Live cell population gated on forward/side scatter plot after LPS/IL4 or LPS stimulation (**h**). n=4 for each genotype in Panel h. **i** Cell division was assayed by CFSE-incorporation. Representative flow cytometry blot and bar graph are showed from three replicates. n=3 for each genotype in Panel i. **j** Representative flow cytometry plots of PI-Annexin V double staining to illustrate the apoptotic populations in indicated genotypes. n, independent mice. Data are represented as mean ± SD. Two-tail unpaired *t*-test was performed for Panel **b** and **i** (upper), one-way ANOVA followed by Dunnett's multiple comparisons test was performed for Panel **d**, **g** and **h** (data from *Rev7* knockout are compared with those from other genotypes), two-tail paired *t*-test was performed for Panel **i** (middle, paired samples are linked with grey lines), ****: $p<0.0001$, ***: $p<0.001$, **: $p<0.01$, *: $p<0.05$, ns: $p>0.05$. P values and defined sample sizes (n) are provided in Supplementary Table 2. Source data are provided as a Source Data file.

Supplementary Figure 2

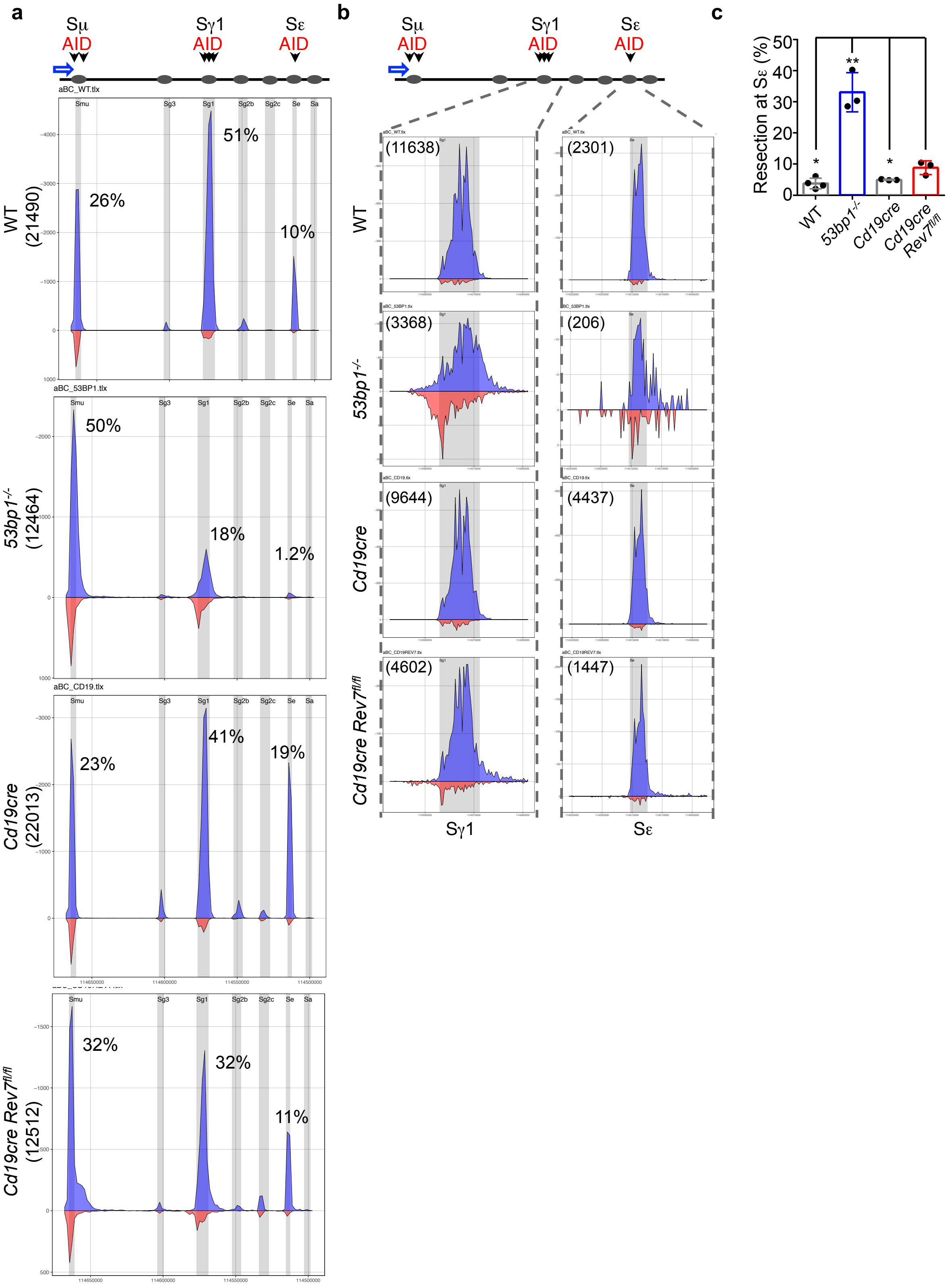

Supplementary Fig. 2. CSR end-joining in REV7 deficiency.

a HTGTS was performed with AID-initiated breaks at Sμ as baits. HTGTS cloning primer are marked with blue arrow (top). Distribution of HTGTS junctions are plotted for each genotype. Zoom-in view of Sγ1 and Sε regions are showed in Panel **b**. Total junction numbers analyzed in each panel are label in parentheses, and percentages of Sμ-Sγ1 and Sμ-Sε junctions in the *IgH* locus are labeled. Data are summary of three independent replicates. **c** End resection of AID-initiated breaks at Sε is showed for indicated B cells. n=4 mice for WT and n=3 mice for the other genotypes. Data are represented as mean ± SD. Two-tail unpaired *t*-test was performed for Panel **c**. **: $p<0.01$, *: $p<0.05$, ns: $p>0.05$. P values and defined sample sizes (n) are provided in Supplementary Table 2. Source data are provided as a Source Data file.

Supplementary Figure 3

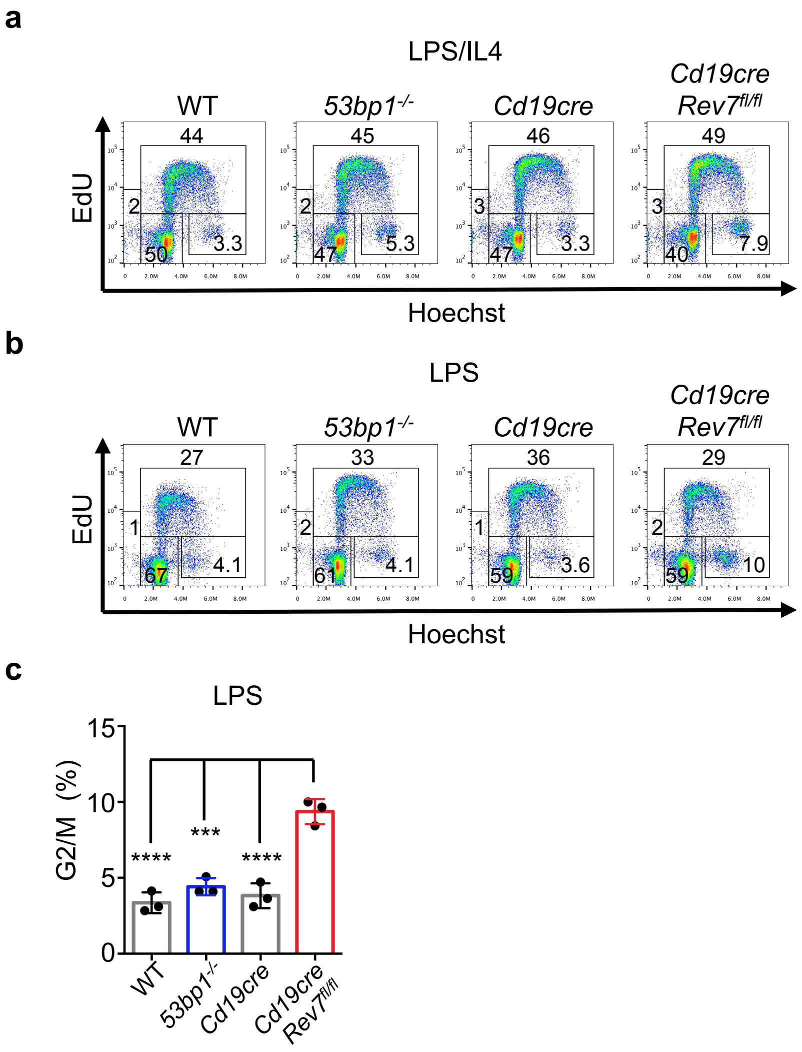

Supplementary Fig. 3. Cell cycle of REV7-deficient B cells.

Cell cycle was assayed by using EdU incorporation assay, and representative flow cytometry blots are showed for LPS/IL4 stimulation (**a**) or LPS stimulation (**b**). **c** Percentage of cells in G2/M phase is plotted for the indicated genotypes upon LPS stimulation. n=3 mice for each genotype. Data are represented as mean ± SD. One-way ANOVA followed by Dunnett's multiple comparisons test was performed for Panel **c**, and data from *Rev7* knockout are compared with those from other genotypes. ****: $p<0.0001$, ***: $p<0.001$. P values and sample sizes are provided in Supplementary Table 2. Source data are provided as a Source Data file.

Supplementary Figure 4

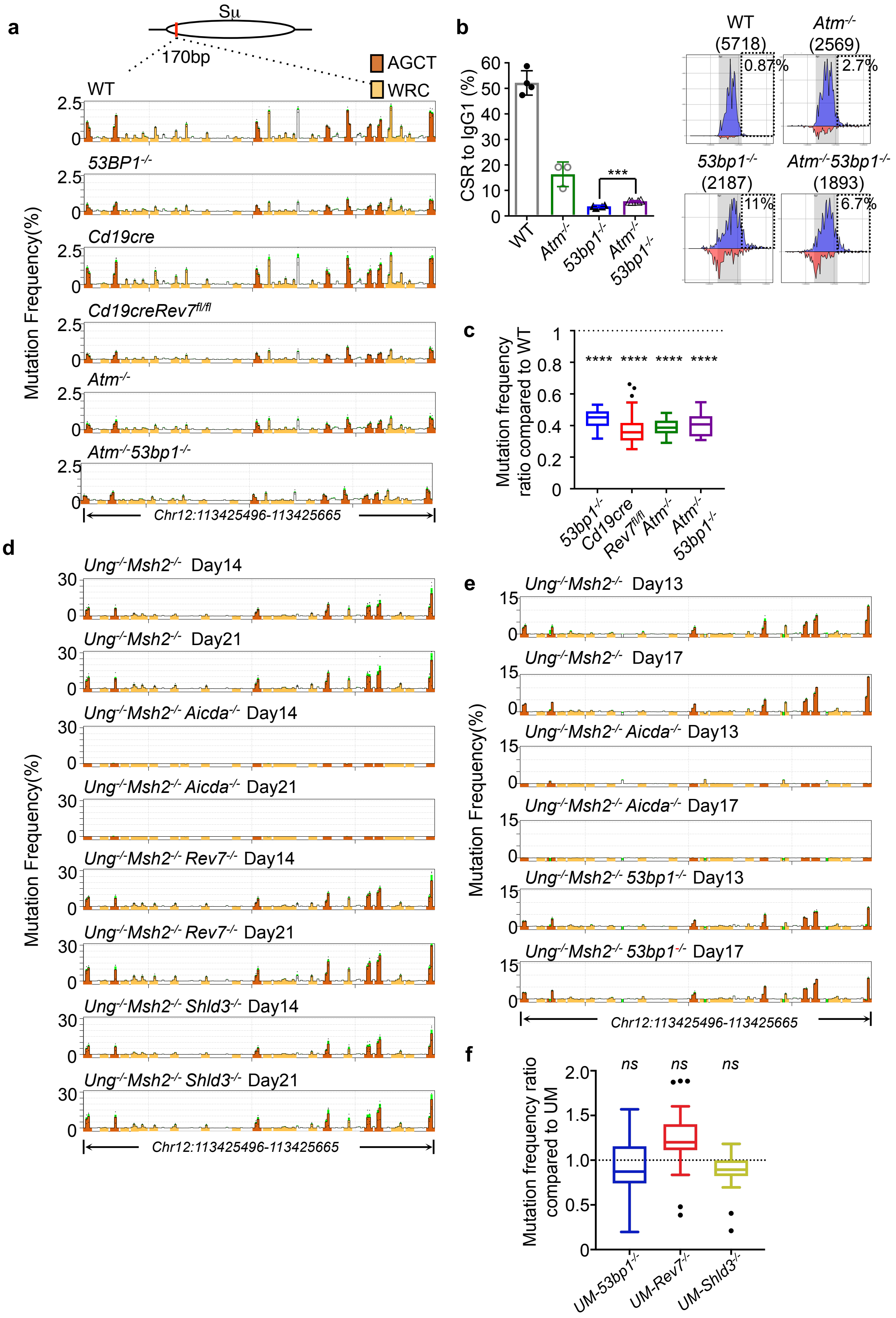

Supplementary Fig. 4. Mutation analysis of 5'Sμ region in CSR-activated B cells.

a Sequence analysis of a 5'Sμ region in indicated CSR-activated B cells. Mutation profiles, i.e. mutation frequency at each nucleotide along a 170 bp 5' Sμ fragment, are showed. Mutate frequency are plotted as the mean percentage of sequences that contain mutation at the indicated nucleotide from three independent experiments. Green bars indicate SEM and data points are showed as black dots. Positions of AGCT and WRC (W: A/T; R: A/G) motifs are marked with orange and yellow bars, respectively. Genomic location (mm10) is labeled at bottom. n=4 independent experiments for each genotype. **b** CSR levels to IgG1 (left) and end resection of Sγ1 region (right) of indicated cell lines. n=3 mice for *Atm*$^{-/-}$ and n=4 mice for the other genotypes. Data are represented as mean ± SD. Two-tail unpaired *t*-test was performed for Panel b. ***: $p<0.001$. **c** Mutation frequency of each nucleotide is normalized to the same nucleotide in control sample, and the ratios of mutation frequencies are plotted as Tukey's box plot. Data are from three independent sets of mice. Nucleotides, with mutation frequency higher than 0.001, were subjected to mutation analysis. Two-tail one sample *t*-test are performed with theoretical mean defined as 1. ****: $p<0.0001$. **d** Mutation profiles of the same 5' Sμ fragment in indicated CH12F3 cell lines after 14- and 21-days culturing with present of CIT stimulation. Panel is labeled as **a**. n=5 for *Ung*$^{-/-}$*Msh2*$^{-/-}$; n=3 for *Ung*$^{-/-}$*Msh2*$^{-/-}$*Aicda*$^{-/-}$; n=4 for the other genotypes. Data are represented as mean ± SEM. **e** Mutation profiles of the same 5' Sμ fragment in indicated CH12F3 cell lines after 13- and 17-days culturing with present of CIT stimulation. Panel is labeled as **a**. n=3 independent experiments for each genotype. **f** Mutation frequency of each nucleotide is normalized to the same nucleotide in control sample, and the ratios of mutation frequencies are plotted as Tukey's box plot. The Tukey's box plot shows the maximum/minimum data point, median, and 75th and 25th quartiles with outliers indicated as points for Panels **c** and **f**. Two-tail one sample *t*-test are performed with theoretical mean defined as 1. Data are from three repeats. ns: $p>0.05$. P values and sample sizes are provided in Supplementary Table 2. Source data are provided as a Source Data file.

Supplementary Figure 5

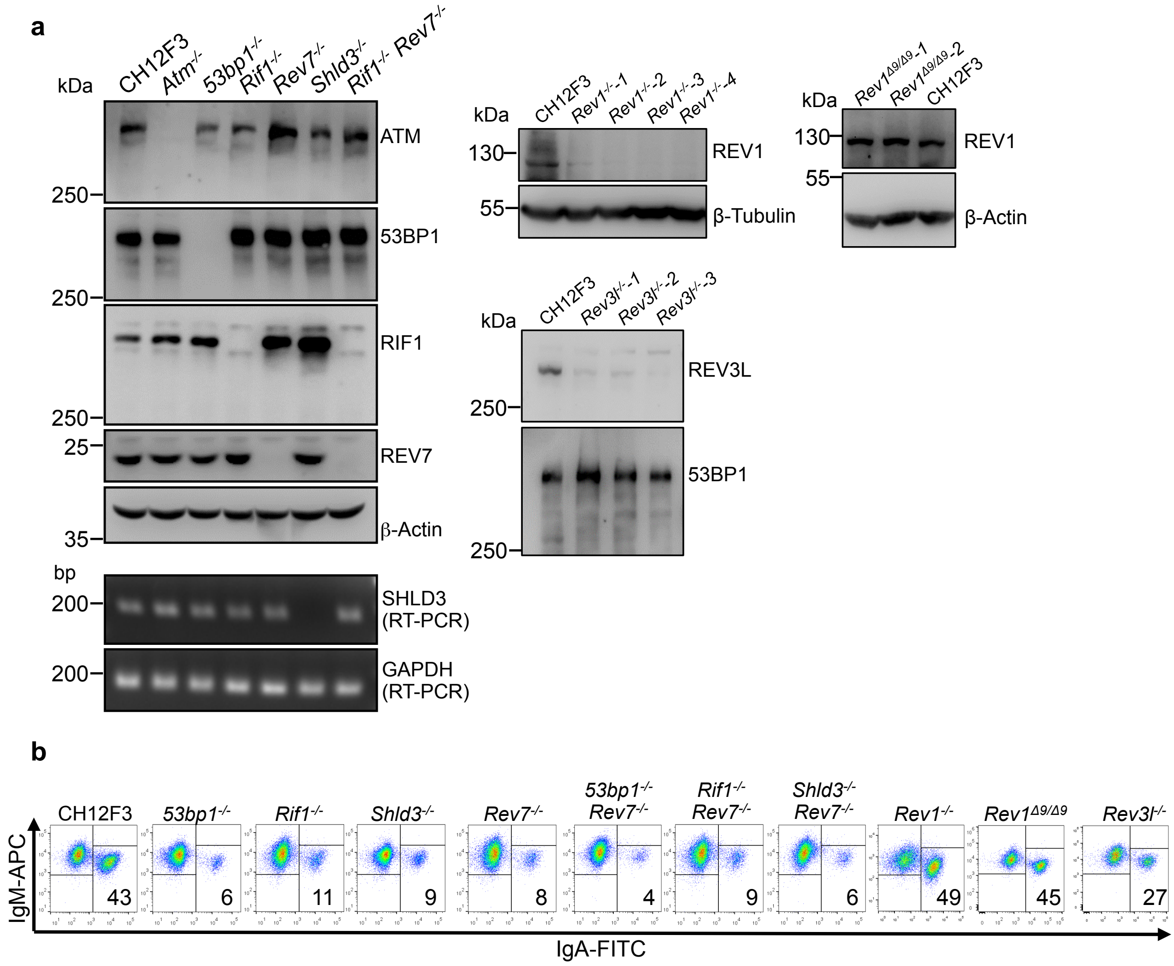

Supplementary Fig. 5. Gene deletion CH12F3 cells.

a Representative western blots of ATM, 53BP1, RIF1, REV7, REV1 and REV3L proteins, and RT-PCR of *Shld3* mRNA in the indicated genotypes. Experiments were independent repeated three times with three or more than three independent clones, and similar results were obtained. **b** Representative flow cytometry blot of CSR to IgA in indicated CH12F3 cell lines.

Supplementary Figure 6

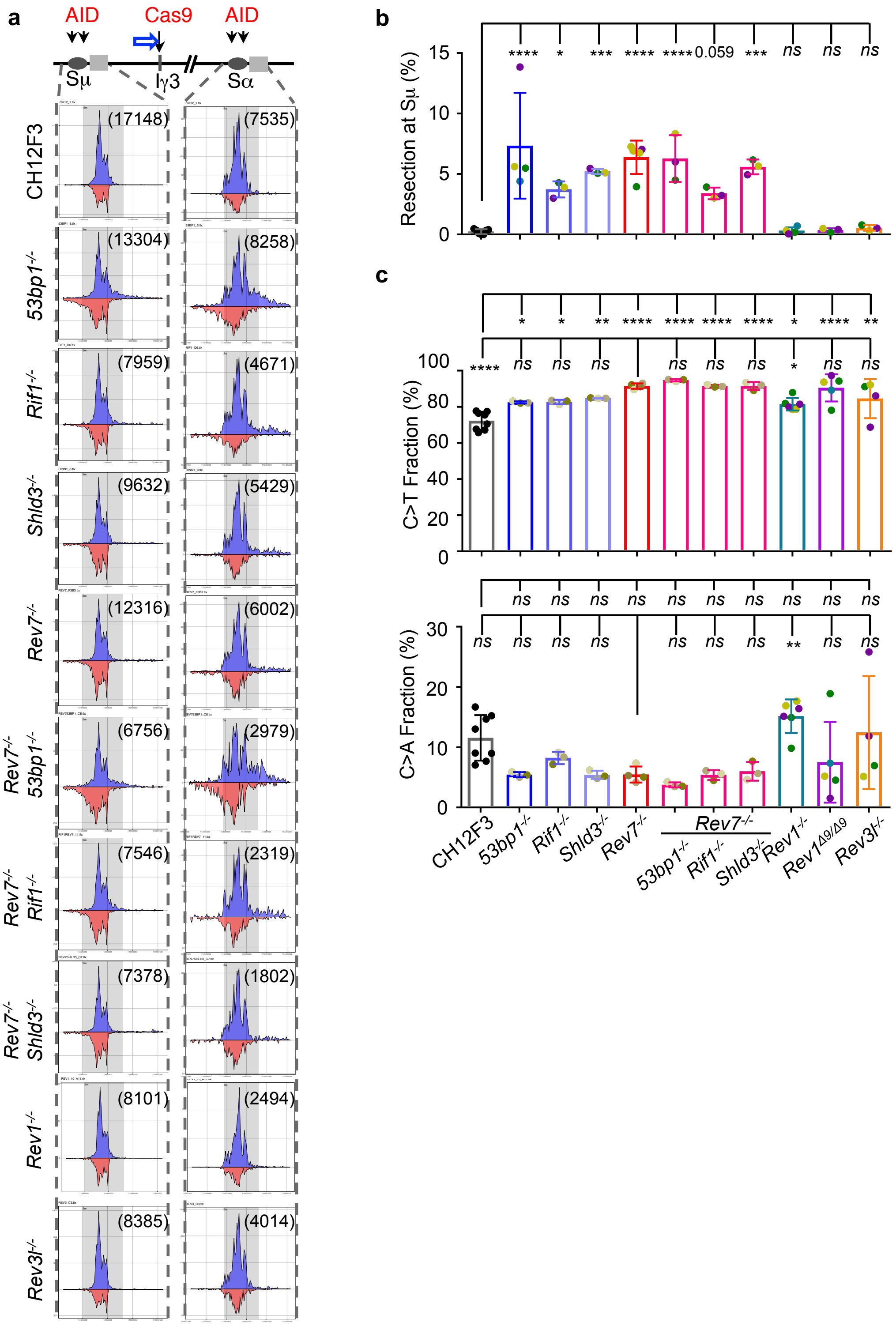

Supplementary Fig. 6. CSR end-joining and mutation spectrum in CH12F3 cells.

a HTGTS was performed with Cas9-initiated breaks at Iγ3 as bait. HTGTS cloning primer are marked with blue arrow (top). Distribution of HTGTS junctions at Sμ and Sα regions are plotted for each genotype. Total junction numbers analyzed in each panel are label in parentheses. **b** Resection level at Sμ is showed for the indicated genotypes. Data are represented as mean ± SD. **c** Mutation spectrum of C/G are plotted. In Panel **b**, n=7 for parental CH12F3 cells; n=5 for *Rev7*$^{-/-}$; n=4 for *53bp1*$^{-/-}$ and *Rev1*$^{-/-}$; n=3 for the other genotypes. In Panel **c**, n=8 for parental CH12F3 cells; n=4 for *Rev7*$^{-/-}$ and *Rev3l*$^{-/-}$; n=6 for *Rev1*$^{-/-}$; n=5 for *Rev1*$^{\Delta 9/\Delta 9}$, n=3 for the other genotypes. Three or more independent clones for each genotype were assayed, and n represents independent experiments. Data are represented as mean ± SD. One-way ANOVA followed by Dunnett's multiple comparisons test was performed for Panel **b**-**c**. Data in knockouts are compared with those in parental CH12F3 cells for all panels. In Panel **c**, an extra comparison is showed by using *Rev7*$^{-/-}$ as the reference group to highlight the difference between DSBR-deficiencies and TLS-deficiencies. ****: p<0.0001, ***: p<0.001, **: p<0.01, *: p<0.05, ns: p>0.05. P values and sample sizes are provided in Supplementary Table 2. Source data are provided as a Source Data file.

Supplementary Figure 7

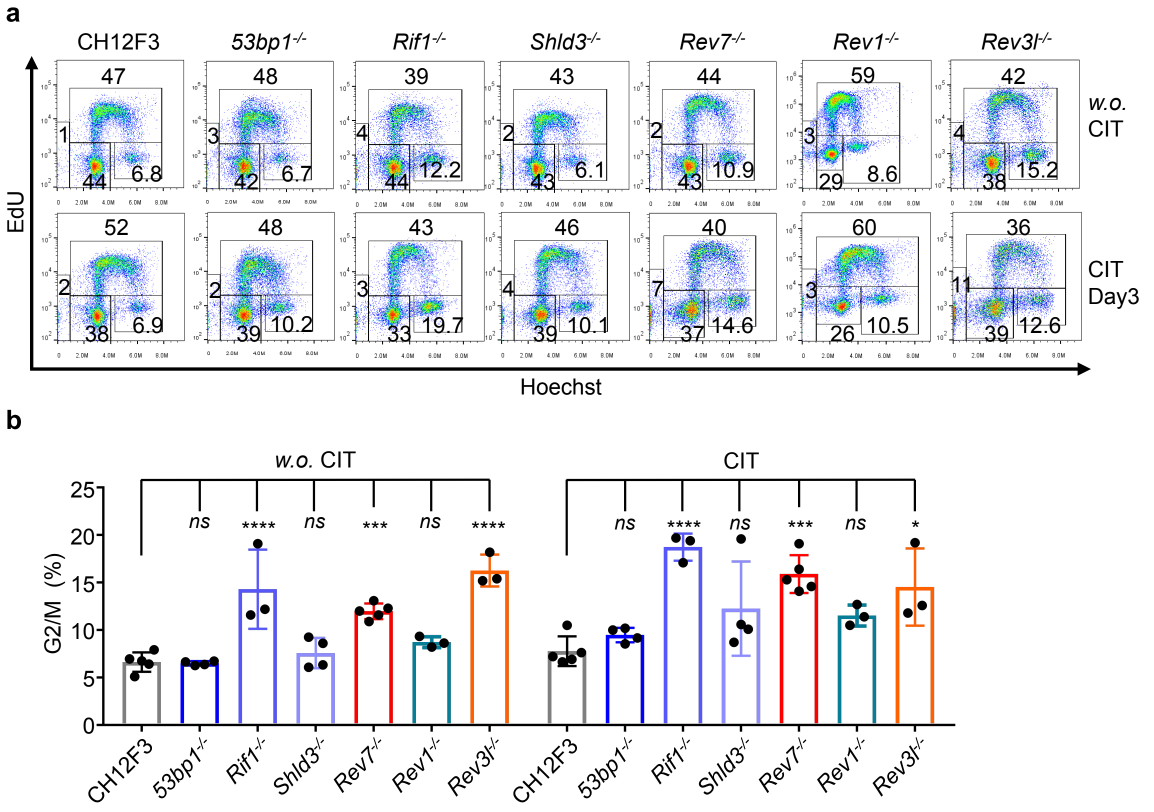

Supplementary Fig. 7. Cell cycle of indicated CH12F3 cells.

Cell cycle of CH12F3 cells was examined by using EdU incorporation assay. Representative flow cytometry blots are showed in Panel **a**, and percentages of G2/M cells are summarized in Panel **b**. In Panel **b**, n=5 for parental CH12F3 cells and *Rev7*$^{-/-}$; n=4 for *53bp1*$^{-/-}$ and *Shld3*$^{-/-}$; n=3 for the other genotypes. Three or more independent clones for each genotype were assayed, and n represents independent experiments. Data are represented as mean ± SD. One-way ANOVA followed by Dunnett's multiple comparisons test was performed for Panel **b**. ****: p<0.0001, ***: p<0.001, *: p<0.05, ns: p>0.05. P values and sample sizes are provided in Supplementary Table 2. Source data are provided as a Source Data file.

Supplementary Figure 8

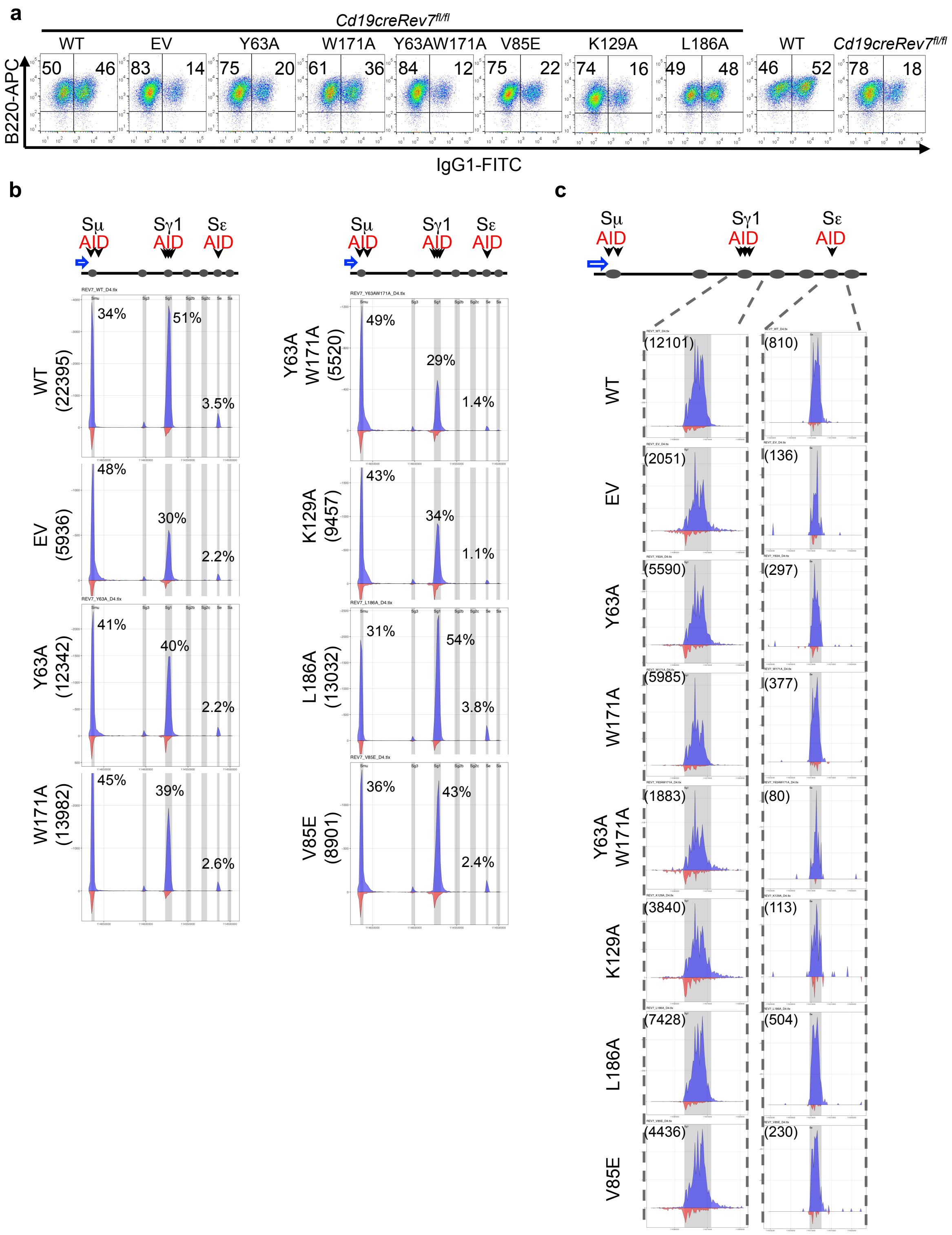

Supplementary Fig. 8. CSR end-joining in *Rev7* knockout B cells complemented with REV7 and its mutants.

a Representative flow cytometry plots of CSR to IgG1 in *Rev7* knockout B cells complemented with REV7 and its mutants. **b** HTGTS was performed with AID-initiated breaks at Sμ as baits. HTGTS cloning primer are marked with blue arrow (top). Distribution of HTGTS junctions are plotted for *Rev7* knockout B cells with REV7 or its mutant. EV: empty vector control. Zoom-in view of Sγ1 and Sε regions are showed in Panel **c**. Total junction numbers analyzed in each panel are label in parentheses, and percentages of Sμ-Sγ1 and Sμ-Sε junctions in the *IgH* locus are labeled. Data are summary of three independent replicates. Source data are provided as a Source Data file.

Supplementary Figure 9

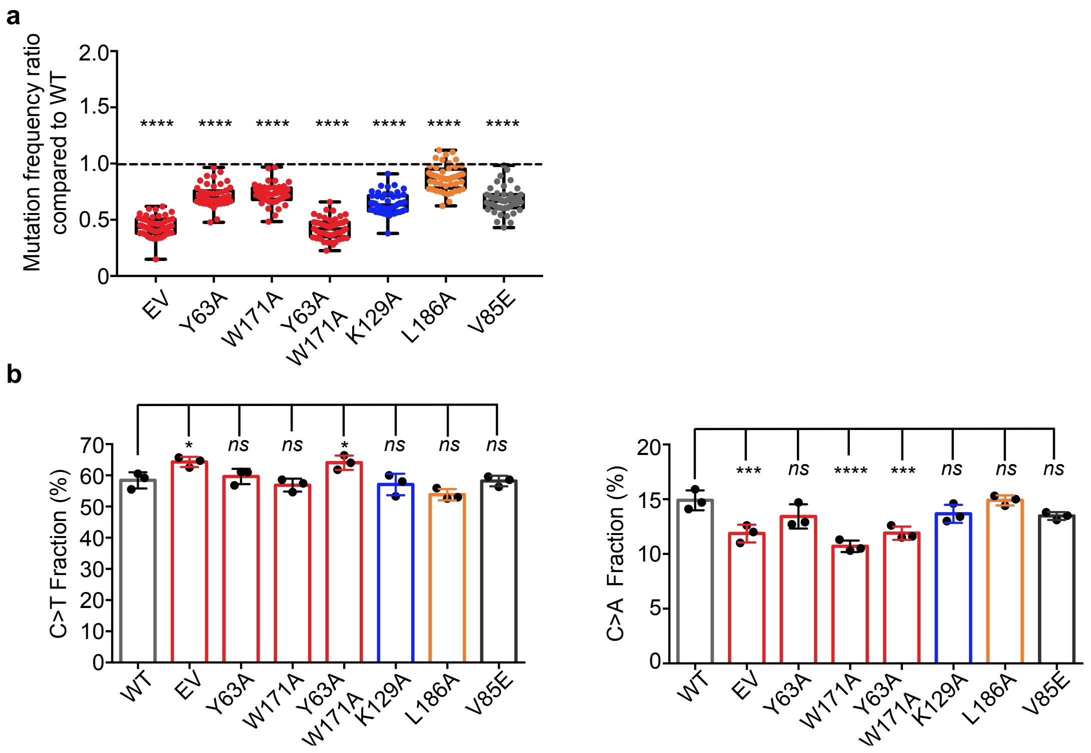

Supplementary Fig. 9. Mutation analysis of 5'Sμ region in *Rev7* knockout B cells complemented with REV7 and its mutants.

a Ratios of mutation frequency are plotted as a box plot. For each position, the mutation frequency in mutant was normalized to that in *Rev7* knockout B cells complemented with wild type REV7. The box and whisker plots show the maximum data point, minimum data point, median value, and 75th and 25th quartiles. Each dot represents relative mutation frequency at one nucleotide and the data are from three independent experiments. Two-tail one sample *t*-test are performed with theoretical mean defined as 1. ****: $p<0.0001$, ns: $p>0.05$. **b** Mutation spectrum analysis in *Rev7* knockout B cells complemented with REV7 and its mutants. n=3 independent experiments. Data are represented as mean ± SD. One-way ANOVA followed by Dunnett's multiple comparisons test was performed for Panel **b**. Data from cells complemented with WT REV7 protein are used as reference group in comparison. ****: $p<0.0001$, ***: $p<0.001$, *: $p<0.05$, ns: $p>0.05$. P values and sample sizes are provided in Supplementary Table 2. Source data are provided as a Source Data file.

Supplementary Figure 10

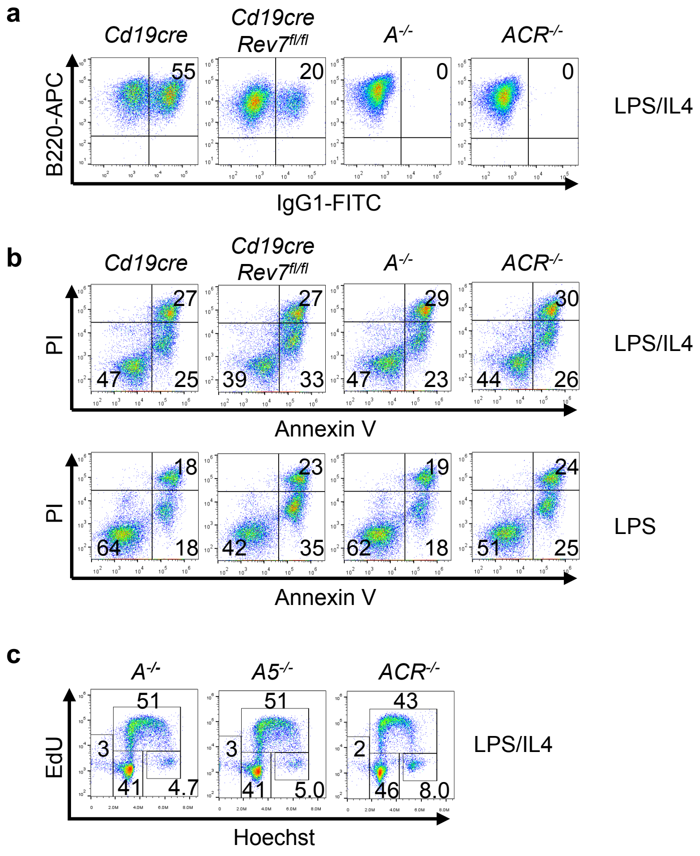

Supplementary Fig. 10. AID and REV7 double-deficient CSR-activated B cells.

Representative flow cytometry plots of CSR to IgG1 (**a**), cell death (**b**), and cell cycle (**c**) of indicated B cells upon LPS/IL4 or LPS stimulation.

Supplementary Figure 11

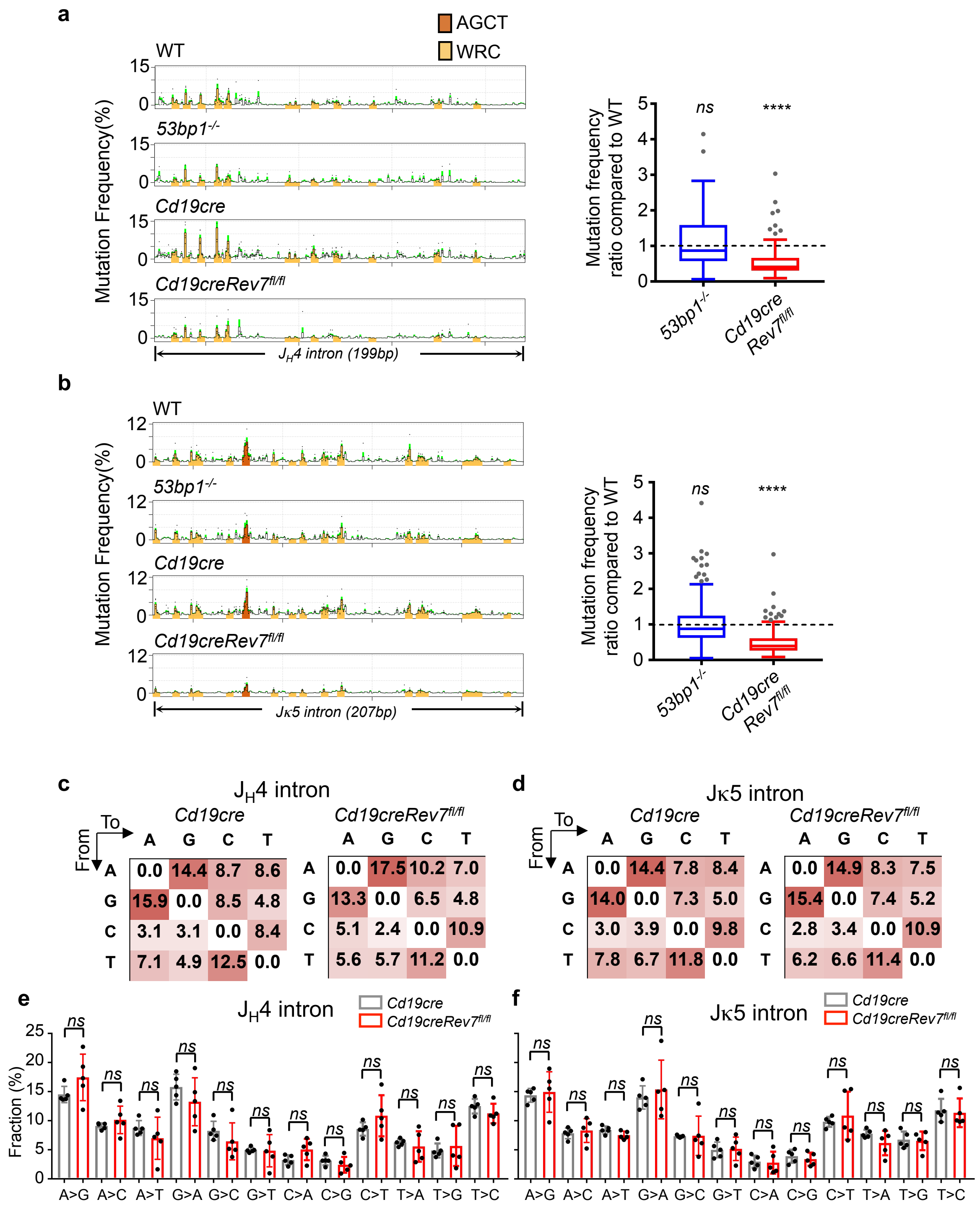

Supplementary Fig. 11. SHM of *Ig V* region introns.

Mutation profiles of J_H4 intron (**a**) and $J\kappa5$ intron (**b**) from Peyer's Patches are showed. Panels are labeled as in Supplementary Fig. 4. Mutate frequency are plotted as the mean percentage of sequences that contain mutation at the indicated nucleotide from four WT mice, four *53bp1*$^{-/-}$ mice, five *Cd19cre* mice and five *Cd19creRev7*$^{fl/fl}$ mice. Nucleotides, with mutation frequency higher than 0.001, were subjected to mutation analysis. Ratio of mutation frequency (*53bp1*$^{-/-}$ vs WT; *Cd19creRev7*$^{fl/fl}$ vs *Cd19cre*) of each single nucleotide in J_H4 intron and $J\kappa5$ intron is plotted as Tukey's box plot at right. The Tukey's box plots show the maximum/minimum data point excluding outliers, median value, and 75th and 25th quartile for Penal **a** right and **b** right, with outliers indicated as points. In Panel **a**, n=4 for WT, n=3 for *53bp1*$^{-/-}$; n=5 for *Cd19cre* and *Cd19cre Rev7*$^{fl/fl}$. In Panel **b**, n=4 for WT, n=4 for *53bp1*$^{-/-}$; n=5 for *Cd19cre* and *Cd19cre Rev7*$^{fl/fl}$. Two-tail one sample *t*-test are performed with theoretical mean defined as 1. ****: $p<0.0001$. Green bars represent SEM. Mutation spectrums of indicated genotype in J_H4 intron (**c**) and $J\kappa5$ intron (**d**), with percentage of total mutations showed as heatmaps. Percentages of specific mutation type in total mutations of J_H4 intron (**e**) and $J\kappa5$ intron (**f**) regions are plotted. In Panels **c**-**f**, n=5 for *Cd19cre* and *Cd19cre Rev7*$^{fl/fl}$. Data are represented as mean ± SD for Panel **e** and **f**. Two-tail unpaired t-test was performed for Panel **e** and **f**. P values and sample sizes are provided in Supplementary Table 2. Source data are provided as a Source Data file.

Supplementary Figure 12

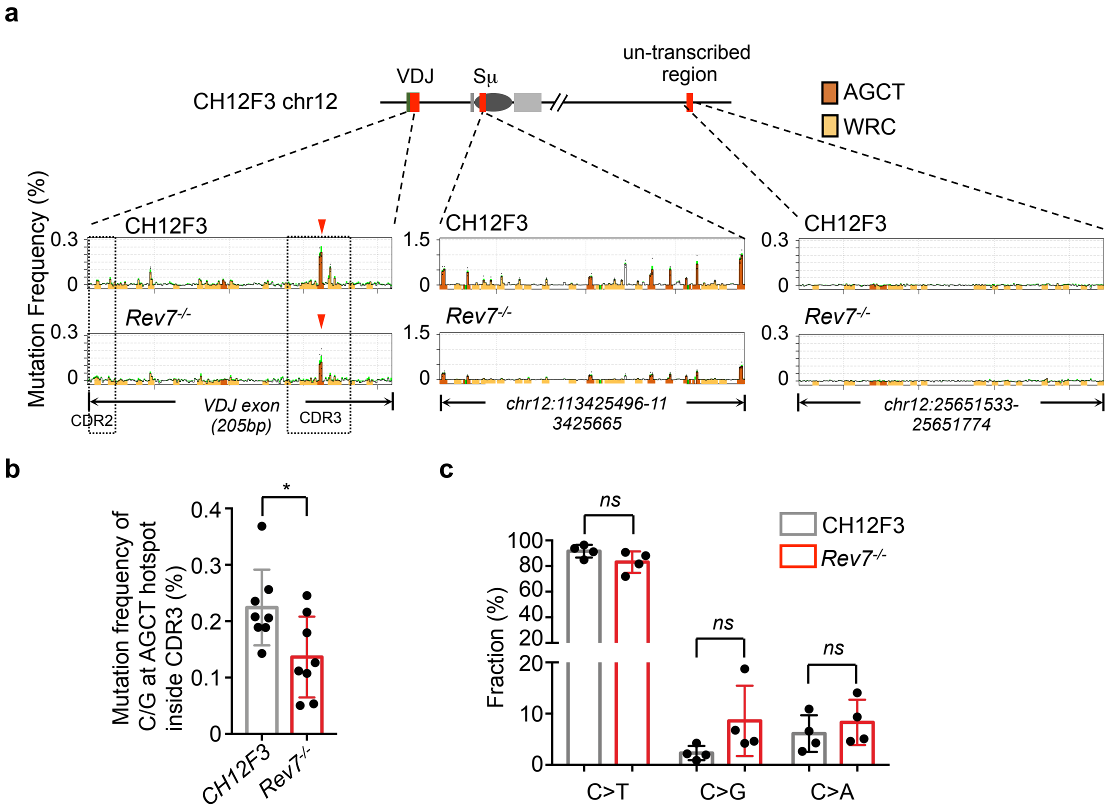

Supplementary Fig. 12. Mutation profiles of three sequences on Chr12 in CH12F3 cells.

a Mutation frequency of three sequences on Chromosome 12 are plotted. Location and annotation are illustrated on top. Mutation profiles are plotted as Supplementary Fig. 4**a**. Complementarity-determining regions (CDRs) are bracketed with dashed lines. The AGCT hotspot inside CDR3 is marked by a red triangle. Green bars represent SEM. **b** Mutation frequency of C/G at the AGCT hotspot inside CDR3. **c** Mutation spectrum of C/G of the AGCT hotspot inside CDR3. n=4 independent experiments for each genotype in Panel **a**, **b** and **c**. Data are represented as mean ± SD in Panel **b** and **c**. Two-tail unpaired t-test was performed for Panel **b** and **c**. *: $p<0.05$, ns: $p>0.05$. P values and sample sizes are provided in Supplementary Table 2. Source data are provided as a Source Data file.

Supplementary Figure 13

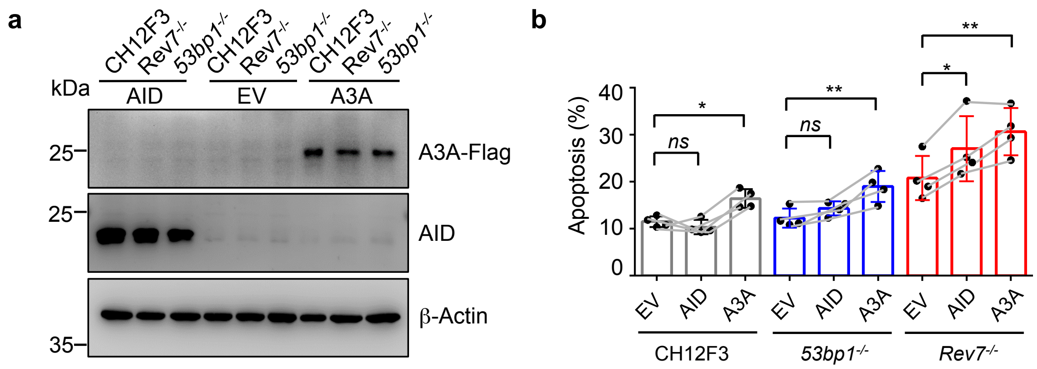

Supplementary Fig. 13. REV7 deficient cells are sensitive to AID/APOBEC-initiated DNA lesions.

a AID and A3A are over-expressed in the indicated cells with representative western blot showed. Experiments were independent repeated twice with similar results obtained. **b** REV7 deficient cells are prone to undergo apoptosis upon AID/APOBEC expression. n=4 independent experiments for each genotype. Data are represented as mean ± SD. Two-tail paired *t*-test was performed for Panel **b**. Paired data are linked with grey lines. **: p<0.01, *: p<0.05, ns: p>0.05. P values and sample sizes are provided in Supplementary Table 2. Source data are provided as a Source Data file.

Supplementary Figure 14

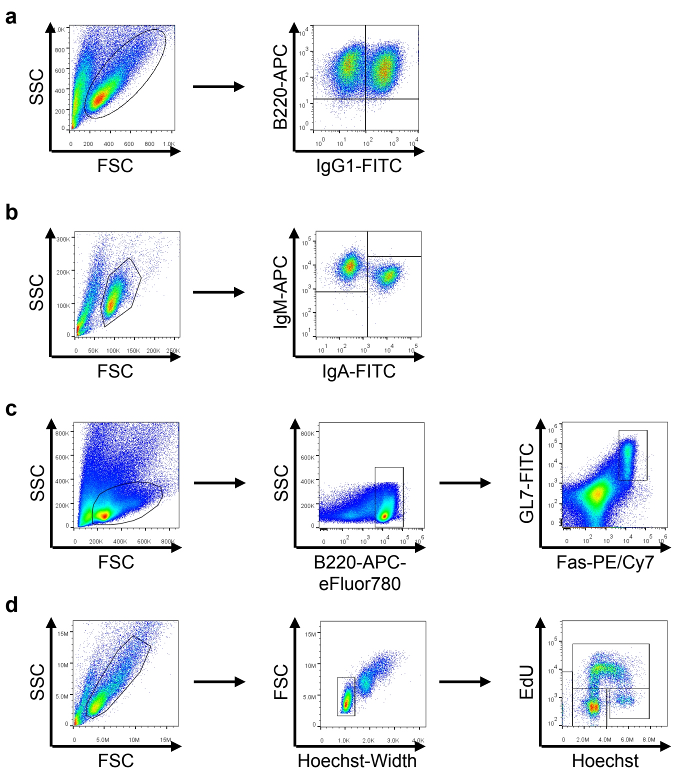

Supplementary Fig. 14. Flow cytometric gating strategies.

a Flow cytometric gating strategy for CSR-activated B cells switching from IgM to IgG showed in Supplementary Fig 1c, 8a and 10a. Data from Supplementary Fig 1c (Sample: *Cd19cre*) are showed as an example.

b Flow cytometric gating strategy for CIT-stimulated CH12F3 cells showed in Supplementary Fig 5b. Data from Supplementary Fig 5b (Sample: CH12F3) are showed as an example.

c Flow cytometric gating strategy for GC B cells showed in Fig 5a, 5b, 5e and 5f. Data from Fig 5b (Sample: *Cd19cre*) are showed as an example.

d Flow cytometric gating strategy of cell cycle showed in Supplementary Fig 3a, 3b, 7a and 10c. Data from Supplementary Fig 7a (Sample: CH12F3 with CIT-stimulation) are showed as an example.

Supplementary Table 1. Primers used in this study.

**Primer Name**	**Sequence 5'->3'**	**Purpose**
sgRNA sequence		
ATM-57	GTCCTCAGTCGATTATCACT	*Atm KO sgRNAs*
ATM-58	TATCTTGATAAACGAGCAGT	
53BP1-192	GGCTGAGAGACTTGTTCCAC	*53bp1 KO sgRNAs*
53BP1-19	CATTCCTGACACTCTACGAT	
53BP1-202	GTTCCAGTGGTCCGAGAAGG	
53BP1-20	CCACGCCCACGAGGTGTAAC	
RIF1-1	TGGAAGTGATACTAAGTAA	*Rif1 KO sgRNAs*
RIF1-2	GGGAGAAGAGTCTGTGAGG	
SHLD3-1	GGAAGTTTGGACTCATCGTA	*Shld3 KO sgRNAs*
SHLD3-2	GGAGTTCATCGCGCCTGATA	
SHLD3-3	GCAGACCAATGCGGCACATT	
SHLD3-4	GCTGGCAAGCGAAACACTCC	
REV7-1	TAGGGATCTGAGCCTGCGG	*Rev7 KO sgRNAs*
REV7-2	CTGGGTGGTGAGAGACAGG	
REV1-Δ10-1	AGTGAGTCATAGGGG	*Rev1 Exon10 KO sgRNAs*
REV1-Δ10-2	AGCTATCAGGACAAAGAGA	
REV1-Δ10-4	GTCTTTGATTTCAATACGG	
REV1-Δ10-5	TACTAACCAATTCCAACAG	
REV1-Δ9-1	TTGTTAACCCCTGATGAGA	*Rev1 Exon9 KO sgRNAs*
REV1-Δ9-2	AGTGTTTCATACATTGCCT	
REV1-Δ9-3	CAGAGGGAAGGAGCACAGG	
REV3-1	TAAAATATGGTTAACACAG	*Rev3 KO sgRNAs*
REV3-2	CGTATGGCACATAGAGGTA	
REV3-3	TTGAACACATGCTGAGCAG	
Genotyping primers in CH12F3		
ATM F2	AGTGTCCGGAAGGTGTGTTC	*Atm KO brand:1172 bp*
ATM R2	GGCCATGACATCCAATCTCT	
53BP1-F1	GGGTTTCACTGGCTTGACTG	*53bp1 KO brand :946 bp*
53BP1-R2	GATGGGGTCACACAGGAGAA	
RIF1-F2	CACGTTCTGAACAGCTCCAT	*Rif1 KO brand:598 bp*
RIF1-R2	TGCCCTCCTAGCTCTCTCTA	
SHLD3-F2	TGAAAACGATCCCAAACAGCT	*Shld3 KO brand:491 bp*
SHLD3-R1	GGAATGTTCCTGAATTGACTGAC	
REV7-F1	TTCTTACCGTTCCCTGTCCC	*Rev7 KO brand:923 bp*
REV7-R1	ACCACTGCCCGGCTAATAAA	
REV1-Δ10-F1	TCCATTTGCCACTTTGAAGC	*Rev1 Exon10 KO brand:551 bp*
REV1-Δ10-R2	AACTTATTATTCAGCTTGGCGAG	
REV1-Δ9--F2	CTCCTGCCCAGAGTGATGTC	*Rev1 Exon9 KO brand:589 bp*
REV1-Δ9--R1	AACCCAGGCTCACATAGTCC	
REV3-F2	ATGAGGTACCTGAAACTTGTCA	*Rev3 KO brand:560 bp*
REV3-R2	CTCTCTTAACACAGGCCCAAAG	
**Q-PCR primers for *Shld3* and *Rev7* flox allele**		
SHLD3-qPCR-F1	TCTGAAGAGGCTGCTGAGAG	*Q-RTPCR primers for Shld3*
SHLD3-qPCR-R1	TTGGTCTGCTCCTTCACTGT	
SHLD3-qPCR-F2	GCGTGAAGCAGTACTTAGCC	*Q-RTPCR primers for Shld3*
SHLD3-qPCR-R2	AGGATCTCTGCTTGTGCTGT	
SHLD3-qPCR-F3	ACACACAGTGAAGGAGCAGA	*Q-RTPCR primers for Shld3*
SHLD3-qPCR-R3	AGGAGTGCAAATGTAGTGTCCT	
REV7-mQRT-F1	GGTGTGTGCCACCATACTTG	

REV7-mQRT-R1	ACAGCCATTCCTAGCTCTCC	*Q-PCR primers Rev7 flox allele*
mGAPDH-RT-F	TGGCAAAGTGGAGATTGTTGCC	*Reference control gene*
mGAPDH-RT-R	AAGATGGTGATGGGCTTCCCG	
Mutation analysis of S region in CH12F3 and CSR-activated B cells		
CH12Sμ_f_hiseq	GACCCAGACAGAGAAAGCCAGACTC	S region first round PCR
CH12Sμ_r_hiseq	GCTACTCCAGAGTAGCTCATTTCAGATC	
CH12Sμ_f2	TTCCCTACACGACGCTCTTCCGATCTNNNNNNAAATGAAGTAGACTGTAATGAACTGGAATG	S region second round PCR, "NNNNNN" represents barcode sequence
CH12Sμ_r2	AGTTCAGACGTGTGCTCTTCCGATCTNNNNNNGCTACTCCAGAGTAGCTCATTTCAGATC	
shm25hi-Fp5	TTCCCTACACGACGCTCTTCCGATCTNNNNNNTGGAAGTGAGATTCAGGCCA	Reference control region first round PCR, "NNNNNN" represents barcode sequence
shm25hi-Rp7	AGTTCAGACGTGTGCTCTTCCGATCTNNNNNNAGTGTTCCCTCCGATTCAGG	
P5_index	AATGATACGGCGACCACCGAGATCTACACTCTTTCCCTACACGAC	Illumina tagging PCR
P7_index	CAAGCAGAAGACGGCATACGAGATNNNNNNGTGACTGGAGTTCAGACGTGT	Illumina tagging PCR, "NNNNNN" represents barcode sequence
Mutation analysis of VDJ region in CH12F3 cells		
CH12VDJ_f	CAGGTCCAACTGCAGCAGCC	VDJ region first round PCR
CH12VDJ_r	GAGAGGTTGTAAGGACTCAC	
CH12VDJ_f2	TTCCCTACACGACGCTCTTCCGATCTNNNNNNGGCCTTGAGTGGATTGGAAATATTA	VDJ region second round PCR, "NNNNNN" represents barcode sequence
CH12VDJ_r2	AGTTCAGACGTGTGCTCTTCCGATCTNNNNNNGAGAGGTTGTAAGGACTCACTGAGGAG	
P5_index	AATGATACGGCGACCACCGAGATCTACACTCTTTCCCTACACGAC	Illumina tagging PCR
P7_index	CAAGCAGAAGACGGCATACGAGATNNNNNNGTGACTGGAGTTCAGACGTGT	Illumina tagging PCR, "NNNNNN" represents barcode sequence
Mutation analysis of J_H4 and Jk5 intron regions in GC B cells		
J558	GGAATTCGCCTGACATCTGAGGACTCTGC	JH4 intron first round PCR
iEur	GACTAGTCCTCTCCAGTTTCGGCTGAATCC	
Vk	GGCTGCAGSTTCAGTGGCAGTGGRTCWGGRAC	Jk5 intron first round PCR, S(GC), R(AG), W(AT)
Jk5	AGCGAATTCAACTTAGGAGACAAAAGAGAGAAC	
JH4Fp5	TTCCCTACACGACGCTCTTCCGATCTNNNNNNGTAAGAATGGCCTCTCCAGGTC	JH4 intron second round PCR, "NNNNNN" represents barcode sequence
JH4R240p7	AGTTCAGACGTGTGCTCTTCCGATCTNNNNNNAATGAGCCTCCAAAGTCCCT	
Jk5Fp5	TTCCCTACACGACGCTCTTCCGATCTNNNNNNTTCAGAAAATCTTGAGAAAATGGAGAG	Jk5 intron second round PCR, "NNNNNN" represents barcode sequence
Jk5R255p7	AGTTCAGACGTGTGCTCTTCCGATCTNNNNNNTCCTCATCCCCTCCAAATCTC	

P5_index	AATGATACGGCGACCACCGAGATCTACA CTCTTTCCCTACACGAC	Illumina tagging PCR
P7_index	CAAGCAGAAGACGGCATACGAGATNNNN NNGTGACTGGAGTTCAGACGTGT	Illumina tagging PCR, "NNNNNN" represents barcode sequence
HTGTS primers		
5'-Bio-Iμ	/5bio/CAGACCTGGGAATGTATGGT	Bio-primer for HTGTS
5'-RED-Iμ	CACACAAAGACTCTGGACCTC	RED-primer for HTGTS
Iγ3 SaCas9 sgRNA	gAAATCTGCAGGACTAACAAAG	sgRNA for producing Iγ3 bait for HTGTS
5'-Iγ3-Bio	/5bio/AGCTGGCAGGACCAAATTCG	Bio-primer for Iγ3 bait
5'-Iγ3-RED	CTGAGTCAGCAGAGAAGAGGT	RED-primer for Iγ3 bait
Primers used for IgH Constant gene germ line transcript		
ImF	CTCTGGCCCTGCTTATTGTTG	μ germ line transcript PCR
CmR	GAAGACATTTGGGAAGGACTGACT	
IaF	CCTGGCTGTTCCCCTATGAA	α germ line transcript PCR
CaR	GAGCTGGTGGGAGTGTCAGTG	
Ig1F	GGC CCTTCCAGATCTTTGAG	γ1 germ line transcript PCR
Cg1R	GGATCC AGAGTTCCAGGTCACT	
Ig3F	TGGGCAAGTGGATCTGAACA	γ3 germ line transcript PCR
Cg3R	CTCAGGGAAGTAGCCTTTGACA	
GAPDHf	ACCCAGAAGACTGTGGATGG	Reference control gene
GAPDHr	AGGAGACAACCTGGTCCTCA	

Supplementary Table 2. Detailed statistical information for all significance assessment performed.

Figure	Relevant information	Statistical test and *p* value	N (independent replicates)	F values or t-values and degrees of freedom
1a(left)		One-way ANOVA<br>*p* value compared to Cd19creRev7$^{fl/fl}$		F(DFn, DFd)<br>F(3, 14)=75.9
1a(left)	WT	<0.0001	5	
1a(left)	*53bp1*$^{-/-}$	0.15	5	
1a(left)	*Cd19cre*	<0.0001	4	
1a(right)		One-way ANOVA<br>*p* value compared to Cd19creRev7$^{fl/fl}$		F(DFn, DFd)<br>F(3, 14)=327.1
1a(right)	WT	<0.0001	5	
1a(right)	*53bp1*$^{-/-}$	0.0002	5	
1a(right)	*Cd19cre*	<0.0001	4	
1b		Unpaired two-tailed *t*-test<br>*p* value compared to Cd19creRev7$^{fl/fl}$		
1b(left)	*Cd19cre*	<0.0001	4	t=3.3, df=6
1b(right)	*Cd19cre*	0.0172	4	T=10.2, df=6

1c(up)		One-way ANOVA<br>*p* value compared to Cd19creRev7$^{fl/fl}$		F(DFn, DFd)<br>F(3, 10)=4.9
1c(up)	WT	0.19	4	
1c(up)	*53bp1*$^{-/-}$	0.99	4	
1c(up)	*Cd19cre*	0.022	3	
1c(down)		One-way ANOVA<br>*p* value compared to Cd19creRev7$^{fl/fl}$		F(DFn, DFd)<br>F(3, 8)=37.8
1c(down)	WT	<0.0001	4	
1c(down)	*53bp1*$^{-/-}$	<0.0001	4	
1c(down)	*Cd19cre*	<0.0001	3	
1e		One-way ANOVA<br>*p* value compared to Cd19creRev7$^{fl/fl}$		F(DFn, DFd)<br>F(3, 9)=71.3
1e	WT	0.0004	4	
1e	*53bp1*$^{-/-}$	0.0004	3	
1e	*Cd19cre*	0.0007	3	
1f		One-way ANOVA<br>*p* value compared to Cd19creRev7$^{fl/fl}$		F(DFn, DFd)<br>F(3, 11)=33.6
1f	WT	<0.0001	4	
1f	*53bp1*$^{-/-}$	0.0004	4	
1f	*Cd19cre*	<0.0001	3	
1g(C>T)		One-way ANOVA<br>*p* value compared to Cd19creRev7$^{fl/fl}$		F(DFn, DFd)<br>F(3, 8)=11.9
1g(C>T)	WT	0.020	3	
1g(C>T)	*53bp1*$^{-/-}$	0.0032	3	
1g(C>T)	*Cd19cre*	0.0017	3	
1g(C>G)		One-way ANOVA<br>*p* value compared to Cd19creRev7$^{fl/fl}$		F(DFn, DFd)<br>F(3, 8)=52.7
1g(C>G)	WT	0.0002	3	
1g(C>G)	*53bp1*$^{-/-}$	<0.0001	3	
1g(C>G)	*Cd19cre*	0.0002	3	
1g(C>A)		One-way ANOVA<br>*p* value compared to Cd19creRev7$^{fl/fl}$		F(DFn, DFd)<br>F(3, 8)=9.5
1g(C>A)	WT	0.84	3	
1g(C>A)	*53bp1*$^{-/-}$	0.065	3	
1g(C>A)	*Cd19cre*	0.079	3	
2a(up)		One-way ANOVA<br>*p* value compared to CH12		F(DFn, DFd)<br>F(7, 244)=12.3
2a(up)	*53bp1*$^{-/-}$	0.0001	9	
2a(up)	*Rif1*$^{-/-}$	<0.0001	9	
2a(up)	*Shld3*$^{-/-}$	<0.0001	9	
2a(up)	*Rev7*$^{-/-}$	<0.0001	12	
2a(up)	*Rev1*$^{-/-}$	1.0	4	
2a(up)	*Rev1*$^{\Delta9/\Delta9}$	0.94	4	
2a(up)	*Rev3l*$^{-/-}$	<0.0001	9	
2a(middle)		One-way ANOVA		F(DFn, DFd)

		p value compared to CH12		F(7, 95)=72.7
2a(middle)	*53bp1*$^{-/-}$	0.088	3	
2a(middle)	*Rif1*$^{-/-}$	0.99	3	
2a(middle)	*Shld3*$^{-/-}$	0.54	3	
2a(middle)	*Rev7*$^{-/-}$	<0.0001	6	
2a(middle)	*Rev1*$^{-/-}$	<0.0001	4	
2a(middle)	*Rev1* $^{\Delta9/\Delta9}$	0.80	3	
2a(middle)	*Rev3l*$^{-/-}$	<0.0001	6	
2a(down)		One-way ANOVA<br>*p* value compared to CH12		F(DFn, DFd)<br>F(7, 148)=28.51
2a(down)	*53bp1*$^{-/-}$	0.71	3	
2a(down)	*Rif1*$^{-/-}$	0.91	3	
2a(down)	*Shld3*$^{-/-}$	0.54	3	
2a(down)	*Rev7*$^{-/-}$	<0.0001	3	
2a(down)	*Rev1*$^{-/-}$	<0.0001	9	
2a(down)	*Rev1* $^{\Delta9/\Delta9}$	0.60	3	
2a(down)	*Rev3l*$^{-/-}$	<0.0001	3	
2b		One-way ANOVA<br>*p* value compared to CH12		F(DFn, DFd)<br>F(10, 29)=50.0
2b	*53bp1*$^{-/-}$	<0.0001	4	
2b	*Rif1*$^{-/-}$	<0.0001	3	
2b	*Shld3*$^{-/-}$	<0.0001	3	
2b	*Rev7*$^{-/-}$	<0.0001	4	
2b	*53bp1*$^{-/-}$ *Rev7*$^{-/-}$	<0.0001	3	
2b	*Rif1*$^{-/-}$ *Rev7*$^{-/-}$	<0.0001	4	
2b	*Shld3*$^{-/-}$ *Rev7*$^{-/-}$	<0.0001	3	
2b	*Rev1*$^{-/-}$	0.56	4	
2b	*Rev1* $^{\Delta9/\Delta9}$	1	4	
2b	*Rev3l*$^{-/-}$	<0.0001	3	
2c		One-way ANOVA<br>*p* value compared to CH12		F(DFn, DFd)<br>F(10, 30)=17.6
2c	*53bp1*$^{-/-}$	<0.0001	4	
2c	*Rif1*$^{-/-}$	<0.0001	3	
2c	*Shld3*$^{-/-}$	0.0003	3	
2c	*Rev7*$^{-/-}$	0.0009	5	
2c	*53bp1*$^{-/-}$ *Rev7*$^{-/-}$	<0.0001	3	
2c	*Rif1*$^{-/-}$ *Rev7*$^{-/-}$	<0.0001	3	
2c	*Shld3*$^{-/-}$ *Rev7*$^{-/-}$	0.025	3	
2c	*Rev1*$^{-/-}$	1.0	4	
2c	*Rev1* $^{\Delta9/\Delta9}$	1.0	3	
2c	*Rev3l*$^{-/-}$	1.0	3	
2d		One-way ANOVA<br>*p* value compared to CH12		F(DFn, DFd)<br>F(10, 34)=97.9
2d	*53bp1*$^{-/-}$	<0.0001	3	
2d	*Rif1*$^{-/-}$	<0.0001	3	
2d	*Shld3*$^{-/-}$	<0.0001	3	

2d	*Rev7*$^{-/-}$	<0.0001	4	
2d	*53bp1*$^{-/-}$ *Rev7*$^{-/-}$	<0.0001	3	
2d	*Rif1*$^{-/-}$ *Rev7*$^{-/-}$	<0.0001	3	
2d	*Shld3*$^{-/-}$ *Rev7*$^{-/-}$	<0.0001	3	
2d	*Rev1*$^{-/-}$	<0.0001	6	
2d	*Rev1*$^{\Delta9/\Delta9}$	<0.0001	5	
2d	*Rev3l*$^{-/-}$	<0.0001	4	
2d		One-way ANOVA<br>*p* value compared to *Rev7*$^{-/-}$		F(DFn, DFd)<br>F(10, 34)=97.9
2d	CH12	<0.0001	8	
2d	*53bp1*$^{-/-}$	<0.0001	3	
2d	*Rif1*$^{-/-}$	<0.0001	3	
2d	*Shld3*$^{-/-}$	<0.0001	3	
2d	*53bp1*$^{-/-}$ *Rev7*$^{-/-}$	0.58	3	
2d	*Rif1*$^{-/-}$ *Rev7*$^{-/-}$	1.0	3	
2d	*Shld3*$^{-/-}$ *Rev7*$^{-/-}$	1.0	3	
2d	*Rev1*$^{-/-}$	1.0	6	
2d	*Rev1*$^{\Delta9/\Delta9}$	0.63	5	
2d	*Rev3l*$^{-/-}$	1.0	4	
2e(left)		One-way ANOVA<br>*p* value compared to CH12		F(DFn, DFd)<br>F(7, 18)=2.5
2e(left)	*53bp1*$^{-/-}$	0.63	3	
2e(left)	*Rif1*$^{-/-}$	0.99	3	
2e(left)	*Shld3*$^{-/-}$	0.68	3	
2e(left)	*Rev7*$^{-/-}$	1.0	3	
2e(left)	*Rev1*$^{-/-}$	0.78	3	
2e(left)	*Rev1*$^{\Delta9/\Delta9}$	0.96	3	
2e(left)	*Rev3l*$^{-/-}$	0.24	3	
2e(right)		One-way ANOVA<br>*p* value compared to CH12		F(DFn, DFd)<br>F(10, 34)=97.9
2e(right)	*53bp1*$^{-/-}$	1.0	3	
2e(right)	*Rif1*$^{-/-}$	1.0	3	
2e(right)	*Shld3*$^{-/-}$	0.20	3	
2e(right)	*Rev7*$^{-/-}$	0.0406	3	
2e(right)	*Rev1*$^{-/-}$	0.53	3	
2e(right)	*Rev1*$^{\Delta9/\Delta9}$	0.96	3	
2e(right)	*Rev3l*$^{-/-}$	0.0028	3	
2f(left)		One-way ANOVA<br>*p* value compared to CH12		F(DFn, DFd)<br>F(7, 27)=7.3
2f(left)	*53bp1*$^{-/-}$	1.0	3	
2f(left)	*Rif1*$^{-/-}$	1.0	3	
2f(left)	*Shld3*$^{-/-}$	1.0	3	
2f(left)	*Rev7*$^{-/-}$	0.014	6	
2f(left)	*Rev1*$^{-/-}$	1.0	4	
2f(left)	*Rev1*$^{\Delta9/\Delta9}$	1.0	4	
2f(left)	*Rev3l*$^{-/-}$	<0.0001	4	

2f(right)		One-way ANOVA<br>*p* value compared to CH12		F(DFn, DFd)<br>F(7, 27)=33.4
2f(right)	*53bp1*$^{-/-}$	1.0	3	
2f(right)	*Rif1*$^{-/-}$	0.19	3	
2f(right)	*Shld3*$^{-/-}$	0.0011	3	
2f(right)	*Rev7*$^{-/-}$	<0.0001	6	
2f(right)	*Rev1*$^{-/-}$	0.79	4	
2f(right)	*Rev1* $^{\Delta 9/\Delta 9}$	0.32	4	
2f(right)	*Rev3l*$^{-/-}$	<0.0001	4	
3b		One-way ANOVA<br>*p* value compared to WT		F(DFn, DFd)<br>F(7, 16)=80.44
3b	EV	<0.0001	3	
3b	Y63A	<0.0001	3	
3b	W171A	0.0005	3	
3b	Y63AW171<br>A	<0.0001	3	
3b	K129A	<0.0001	3	
3b	L186A	1.0	3	
3b	V85E	<0.0001	3	
3c		One-way ANOVA<br>*p* value compared to WT		F(DFn, DFd)<br>F(7, 16)=10.38
3c	EV	0.0046	3	
3c	Y63A	0.14	3	
3c	W171A	1.0	3	
3c	Y63AW171<br>A	0.0018	3	
3c	K129A	0.0004	3	
3c	L186A	0.99	3	
3c	V85E	0.0015	3	
3d		One-way ANOVA<br>*p* value compared to WT		F(DFn, DFd)<br>F(7, 16)=14.31
3d	EV	0.025	3	
3d	Y63A	1.0	3	
3d	W171A	0.0026	3	
3d	Y63AW171<br>A	0.074	3	
3d	K129A	0.48	3	
3d	L186A	0.022	3	
3d	V85E	0.67	3	
3e		One-way ANOVA<br>*p* value compared to WT		F(DFn, DFd)<br>F(7, 16)=46.75
3e	EV	<0.0001	3	
3e	Y63A	<0.0001	3	
3e	W171A	0.0054	3	
3e	Y63AW171<br>A	<0.0001	3	
3e	K129A	<0.0001	3	
3e	L186A	1.0	3	
3e	V85E	<0.0001	3	
3f		One-way ANOVA<br>*p* value compared to WT		F(DFn, DFd)<br>F(7, 16)=9.838
3f	EV	0.0001	3	

3f	Y63A	0.92	3	
3f	W171A	0.56	3	
3f	Y63AW171A	0.0002	3	
3f	K129A	0.058	3	
3f	L186A	0.43	3	
3f	V85E	0.018	3	
3g		One-way ANOVA<br>*p* value compared to WT		F(DFn, DFd)<br>F(7, 16)=8.741
3g	EV	0.0003	3	
3g	Y63A	0.87	3	
3g	W171A	0.70	3	
3g	Y63AW171A	0.0007	3	
3g	K129A	0.70	3	
3g	L186A	0.73	3	
3g	V85E	0.0088	3	
4a		Paired two-tailed *t*-test<br>*p* value compared to PNA^{low}		
4a(left)	PNA^{high}	0.013	5	t=4.2, df=4
4a(right)	PNA^{high}	0.0080	5	t=4.9, df=4
4b(up)		One-way ANOVA<br>*p* value compared to $AID^{-/-}$		F(DFn, DFd)<br>F(3, 8)=6.758
4b(up)	*Cd19cre*	0.71	3	
4b(up)	$Cd19creRev7^{fl/fl}$	0.013	3	
4b(up)	$A^{-/-}$	1.0	3	
4b(down)		One-way ANOVA<br>*p* value compared to $AID^{-/-}$		F(DFn, DFd)<br>F(3, 8)=6.321
4b(down)	*Cd19cre*	0.65	3	
4b(down)	$Cd19creRev7^{fl/fl}$	0.046	3	
4b(down)	$A^{-/-}$	0.89	3	
4c(up)		One-way ANOVA<br>*p* value compared to $AID^{-/-}$		F(DFn, DFd)<br>F(3, 8)=43.94
4c(up)	*Cd19cre*	0.15	3	
4c(up)	$Cd19creRev7^{fl/fl}$	<0.0001	3	
4c(up)	$A^{-/-}$	0.82	3	
4c(down)		One-way ANOVA<br>*p* value compared to $AID^{-/-}$		F(DFn, DFd)<br>F(3, 8)=17.27
4c(down)	*Cd19cre*	0.078	3	
4c(down)	$Cd19creRev7^{fl/fl}$	0.015	3	
4c(down)	$A^{-/-}$	0.093	3	
4d		Paired two-tailed *t*-test<br>*p* value compared to Day3		
4d(left)	Day4	0.089	3	t=3.1, df=2
4d(right)	Day4	0.16	3	t=2.1, df=2
4e		One-way ANOVA<br>*p* value compared to $AID^{-/-}$		F(DFn, DFd)<br>F(2, 6)=14.1

4e	*A5*$^{-/-}$	1.0	3	
4e	*ACR*$^{-/-}$	0.0067	3	
5a		Unpaired two-tailed *t*-test<br>*p* value compared to *Cd19cre*		
5a	*Cd19creRev7*$^{fl/fl}$	0.0055	7	t=3.4, df=11
5b		Unpaired two-tailed *t*-test<br>*p* value compared to *Cd19cre*		
5b	*Cd19creRev7*$^{fl/fl}$	0.0012	7	t=4.3, df=11
5d		Paired two-tailed *t*-test<br>*p* value compared to PNA^{low}		
5d(left)	PNA^{high}	0.0021	5	t=7.1, df=4
5d(right)	PNA^{high}	0.0030	5	t=6.4, df=4
5e		Unpaired two-tailed *t*-test<br>*p* value compared to *AID*$^{-/-}$		
5e	*ACR*$^{-/-}$	0.46	5	t=0.79, df=7
5f		Unpaired two-tailed *t*-test<br>*p* value compared to *AID*$^{-/-}$		
5f	*ACR*$^{-/-}$	0.73	6	t=0.35, df=10
6a(CH12)		One-way ANOVA<br>*p* value compared to CH12 control		F(DFn, DFd)<br>F(3, 18)=31.8
6a(CH12)	CIT	0.38	5	
6a(Rev7)		One-way ANOVA<br>*p* value compared to *Rev7*$^{-/-}$ control		F(DFn, DFd)<br>F(3, 18)=31.8
6a(Rev7)	CIT	<0.0001	6	
6a(*Ung*)		One-way ANOVA<br>*p* value compared to *Ung*$^{-/-}$ control		F(DFn, DFd)<br>F(3, 13)=33.4
6a(*Ung*)	CIT	0.42	4	
6a(*UngRev7*)		One-way ANOVA<br>*p* value compared to *Ung*$^{-/-}$ *Rev7*$^{-/-}$ control		F(DFn, DFd)<br>F(3, 13)=33.4
6a(*UngRev7*)	CIT	0.25	6	
6a(*Msh2*)		One-way ANOVA<br>*p* value compared to *Msh2*$^{-/-}$ control		F(DFn, DFd)<br>F(3, 16)=16.0
6a(*Msh2*)	CIT	1.0	6	
6a(*Msh2Rev7*)		One-way ANOVA<br>*p* value compared to *Msh2*$^{-/-}$ *Rev7*$^{-/-}$ control		F(DFn, DFd)<br>F(3, 16)=16.0
6a(*Msh2 Rev7*)	CIT	0.0022	6	
6a(*UM*)		One-way ANOVA<br>*p* value compared to *UM*$^{-/-}$ control		F(DFn, DFd)<br>F(3, 12)=5.6
6a(*UM*)	CIT	0.15	4	
6a(*UMR*)		One-way ANOVA<br>*p* value compared to *UMR*$^{-/-}$ control		F(DFn, DFd)<br>F(3, 12)=5.6
6a(*UMR*)	CIT	0.50	4	
6b(CH12)		One-way ANOVA		F(DFn, DFd)

		p value compared to CH12 control		F(3, 18)=58.9
6b(CH12)	CIT	0.54	6	
6b(*Rev7*)		One-way ANOVA<br>*p* value compared to $Rev7^{-/-}$ control		F(DFn, DFd)<br>F(3, 18)=58.9
6b(*Rev7*)	CIT	<0.0001	6	
6b(*Ung*)		One-way ANOVA<br>*p* value compared to $Ung^{-/-}$ control		F(DFn, DFd)<br>F(3, 16)=19.7
6b(*Ung*)	CIT	0.98	6	
6b(*Ung Rev7*)		One-way ANOVA<br>*p* value compared to $Ung^{-/-}$ $Rev7^{-/-}$ control		F(DFn, DFd)<br>F(3, 16)=19.7
6b(*Ung Rev7*)	CIT	0.18	6	
6b(*Msh2*)		One-way ANOVA<br>*p* value compared to $Msh2^{-/-}$ control		F(DFn, DFd)<br>F(3, 16)=27.03
6b(*Msh2*)	CIT	1.0	6	
6b(*Msh2 Rev7*)		One-way ANOVA<br>*p* value compared to $Msh2^{-/-}$ $Rev7^{-/-}$ control		F(DFn, DFd)<br>F(3, 16)=27.03
6b(*Msh2 Rev7*)	CIT	0.0017	6	
6b(*UM*)		One-way ANOVA<br>*p* value compared to $UM^{-/-}$ control		F(DFn, DFd)<br>F(3, 12)=4.3
6b(*UM*)	CIT	0.33	4	
6b(*UMR*)		One-way ANOVA<br>*p* value compared to $UMR^{-/-}$ control		F(DFn, DFd)<br>F(3, 12)=4.3
6b(*UMR*)	CIT	0.34	4	
Supplementary data figure				
1b		Unpaired two-tailed *t*-test<br>*p* value compared to *Cd19cre*		
1b	$Cd19creRev7^{fl/fl}$	0.34	3	t=1.1, df=4
1d(left)		One-way ANOVA<br>*p* value compared to $Cd19creRev7^{fl/fl}$		F(DFn, DFd)<br>F(3, 14)=42.7
1d(left)	WT	0.0036	5	
1d(left)	$53bp1^{-/-}$	0.031	5	
1d(left)	*Cd19cre*	<0.0001	4	
1d(right)		One-way ANOVA<br>*p* value compared to $Cd19creRev7^{fl/fl}$		F(DFn, DFd)<br>F(3, 14)=133.9
1d(right)	WT	<0.0001	5	
1d(right)	$53bp1^{-/-}$	0.0004	5	
1d(right)	*Cd19cre*	<0.0001	4	
1g(left-Day3)		One-way ANOVA<br>*p* value compared to $Cd19creRev7^{fl/fl}$		F(DFn, DFd)<br>F(3, 12)=2.3
1g(left-Day3)	WT	0.98	4	

1g(left-Day3)	*53bp1*$^{-/-}$	1.0	4	
1g(left-Day3)	*Cd19cre*	0.11	4	
1g(left-Day4)		One-way ANOVA<br>*p* value compared to<br>*Cd19creRev7*$^{fl/fl}$		F(DFn, DFd)<br>F(3, 12)=9.0
1g(left-Day4)	WT	0.0090	4	
1g(left-Day4)	*53bp1*$^{-/-}$	0.037	4	
1g(left-Day4)	*Cd19cre*	0.0008	4	
1g(right-Day3)		One-way ANOVA<br>*p* value compared to<br>*Cd19creRev7*$^{fl/fl}$		F(DFn, DFd)<br>F(3, 14)=2.7
1g(right-Day3)	WT	0.48	5	
1g(right-Day3)	*53bp1*$^{-/-}$	0.70	5	
1g(right-Day3)	*Cd19cre*	0.037	4	
1g(right-Day4)		One-way ANOVA<br>*p* value compared to<br>*Cd19creRev7*$^{fl/fl}$		F(DFn, DFd)<br>F(3, 14)=7.8
1g(right-Day4)	WT	0.0014	5	
1g(right-Day4)	*53bp1*$^{-/-}$	0.020	5	
1g(right-Day4)	*Cd19cre*	0.0042	4	
1h(left-Day3)		One-way ANOVA<br>*p* value compared to<br>*Cd19creRev7*$^{fl/fl}$		F(DFn, DFd)<br>F(3, 8)=1.8
1h(left-Day3)	WT	0.12	3	
1h(left-Day3)	*53bp1*$^{-/-}$	0.55	3	
1h(left-Day3)	*Cd19cre*	0.36	3	
1h(left-Day4)		One-way ANOVA<br>*p* value compared to<br>*Cd19creRev7*$^{fl/fl}$		F(DFn, DFd)<br>F(3, 8)=7.0
1h(left-Day4)	WT	0.027	3	
1h(left-Day4)	*53bp1*$^{-/-}$	0.035	3	
1h(left-Day4)	*Cd19cre*	0.0063	3	
1h(right-Day3)		One-way ANOVA<br>*p* value compared to<br>*Cd19creRev7*$^{fl/fl}$		F(DFn, DFd)<br>F(3, 8)=6.9
1h(right-Day3)	WT	0.048	3	
1h(right-Day3)	*53bp1*$^{-/-}$	0.0055	3	
1h(right-Day3)	*Cd19cre*	0.0495	3	
1h(right-Day4)		One-way ANOVA<br>*p* value compared to<br>*Cd19creRev7*$^{fl/fl}$		F(DFn, DFd)<br>F(3, 14)=12.5
1h(right-Day4)	WT	0.0081	3	
1h(right-Day4)	*53bp1*$^{-/-}$	0.0009	3	
1h(right-Day4)	*Cd19cre*	0.021	3	
1i(up)		Unpaired two-tailed *t*-test<br>*p* value compared to WT		
1i(up)	*Cd19creRev7*$^{fl/fl}$	0.015	3	t=4.1, df=4
1i(middle)		Paired two-tailed *t*-test<br>*p* value compared to WT 0		
1i(middle)	*Cd19creRev7*$^{fl/fl}$ 0	0.16	3	t=2.2, df=2
1i(middle)		Paired two-tailed *t*-test		

		p value compared to WT 1		
1i(middle)	*Cd19creRev7*$^{fl/fl}$ 1	0.20	3	t=1.9, df=2
1i(middle)		Paired two-tailed *t*-test<br>*p* value compared to WT 2		
1i(middle)	*Cd19creRev7*$^{fl/fl}$ 2	0.52	3	t=0.78, df=2
1i(middle)		Paired two-tailed *t*-test<br>*p* value compared to WT 3		
1i(middle)	*Cd19creRev7*$^{fl/fl}$ 3	0.65	3	t=0.53, df=2
1i(middle)		Paired two-tailed *t*-test<br>*p* value compared to WT 4		
1i(middle)	*Cd19creRev7*$^{fl/fl}$ 4	0.53	3	t=0.76, df=2
1i(middle)		Paired two-tailed *t*-test<br>*p* value compared to WT 5		
1i(middle)	*Cd19creRev7*$^{fl/fl}$ 5	0.12	3	t=2.7, df=2
1i(middle)		Paired two-tailed *t*-test<br>*p* value compared to WT 6		
1i(middle)	*Cd19creRev7*$^{fl/fl}$ 6	0.0038	3	t=16.3, df=2
2c		Unpaired two-tailed *t*-test<br>*p* value compared to *Cd19creRev7*$^{fl/fl}$		
2c	WT	0.018	3	t=3.4, df=5
2c	*53bp1*$^{-/-}$	0.0032	3	t=6.3, df=4
2c	*Cd19cre*	0.038	3	t=3.1, df=4
3c		One-way ANOVA<br>*p* value compared to Cd19creRev7$^{fl/fl}$		F(DFn, DFd)<br>F(3, 8)=43.3
3c	WT	<0.0001	3	
3c	*53bp1*$^{-/-}$	0.0001	3	
3c	*Cd19cre*	<0.0001	3	
4b		Unpaired two-tailed *t*-test<br>*p* value compared to *Atm*$^{-/-}$ *53bp1*$^{-/-}$		
4b	*53bp1*$^{-/-}$	0.0002	3	t=7.9, df=6
4c		One sample *t*-test<br>*p* value compared to WT		
4c	*53bp1*$^{-/-}$	<0.0001	3	t=46.4, df=23
4c	*Cd19creRev7*$^{fl/fl}$	<0.0001	4	t=46.2, df=47
4c	*Atm*$^{-/-}$	<0.0001	3	t=55.7, df=23
4c	*Atm*$^{-/-}$*53bp1*$^{-/-}$	<0.0001	3	t=38.5, df=23
4f		One sample *t*-test<br>*p* value compared to UM		
4f	*UM-53bp1*$^{-/-}$	0.54	3	t=0.5 df=37
4f	*UM- Rev7*$^{-/-}$	0.14	4	t=1.4, df=51
4f	*UM- Shld3*$^{-/-}$	0.48	3	t=0.7, df=51
6b		One-way ANOVA		F(DFn, DFd)

		p value compared to CH12		F(10, 30)=12.6
6b	*53bp1*$^{-/-}$	<0.0001	4	
6b	*Rif1*$^{-/-}$	0.028	3	
6b	*Shld3*$^{-/-}$	0.0008	3	
6b	*Rev7*$^{-/-}$	<0.0001	5	
6b	*53bp1*$^{-/-}$ *Rev7*$^{-/-}$	<0.0001	3	
6b	*Rif1*$^{-/-}$ *Rev7*$^{-/-}$	0.059	3	
6b	*Shld3*$^{-/-}$ *Rev7*$^{-/-}$	0.0003	3	
6b	*Rev1*$^{-/-}$	1.0	4	
6b	*Rev1*$^{\Delta9/\Delta9}$	1.0	3	
6b	*Rev3l*$^{-/-}$	1.0	3	
6c(up)		One-way ANOVA<br>*p* value compared to CH12		F(DFn, DFd)<br>F(10, 34)=9.2
6c(up)	*53bp1*$^{-/-}$	0.035	3	
6c(up)	*Rif1*$^{-/-}$	0.029	3	
6c(up)	*Shld3*$^{-/-}$	0.0061	3	
6c(up)	*Rev7*$^{-/-}$	<0.0001	4	
6c(up)	*53bp1*$^{-/-}$ *Rev7*$^{-/-}$	<0.0001	3	
6c(up)	*Rif1*$^{-/-}$ *Rev7*$^{-/-}$	<0.0001	3	
6c(up)	*Shld3*$^{-/-}$ *Rev7*$^{-/-}$	<0.0001	3	
6c(up)	*Rev1*$^{-/-}$	0.014	6	
6c(up)	*Rev1*$^{\Delta9/\Delta9}$	<0.0001	5	
6c(up)	*Rev3l*$^{-/-}$	0.0025	4	
6c(up)		One-way ANOVA<br>*p* value compared to *Rev7*$^{-/-}$		F(DFn, DFd)<br>F(10, 34)=9.2
6c(up)	CH12	<0.0001	8	
6c(up)	*53bp1*$^{-/-}$	0.15	3	
6c(up)	*Rif1*$^{-/-}$	0.16	3	
6c(up)	*Shld3*$^{-/-}$	0.42	3	
6c(up)	*53bp1*$^{-/-}$ *Rev7*$^{-/-}$	0.97	3	
6c(up)	*Rif1*$^{-/-}$ *Rev7*$^{-/-}$	1.0	3	
6c(up)	*Shld3*$^{-/-}$ *Rev7*$^{-/-}$	1.0	3	
6c(up)	*Rev1*$^{-/-}$	0.026	6	
6c(up)	*Rev1*$^{\Delta9/\Delta9}$	1.0	5	
6c(up)	*Rev3l*$^{-/-}$	0.31	4	
6c(down)		One-way ANOVA<br>*p* value compared to CH12		F(DFn, DFd)<br>F(10, 34)=3.5
6c(down)	*53bp1*$^{-/-}$	0.22	3	
6c(down)	*Rif1*$^{-/-}$	0.83	3	
6c(down)	*Shld3*$^{-/-}$	0.22	4	
6c(down)	*Rev7*$^{-/-}$	0.15	3	
6c(down)	*53bp1*$^{-/-}$ *Rev7*$^{-/-}$	0.066	3	

6c(down)	*Rif1*$^{-/-}$ *Rev7*$^{-/-}$	0.22	3	
6c(down)	*Shld3*$^{-/-}$ *Rev7*$^{-/-}$	0.31	3	
6c(down)	*Rev1*$^{-/-}$	0.54	6	
6c(down)	*Rev1* $^{\Delta 9/\Delta 9}$	0.47	5	
6c(down)	*Rev3l*$^{-/-}$	1.0	4	
6c(down)		One-way ANOVA<br>*p* value compared to *Rev7*$^{-/-}$		F(DFn, DFd)<br>F(10, 34)=3.5
6c(down)	CH12	0.14	3	
6c(down)	*53bp1*$^{-/-}$	1.0	3	
6c(down)	*Rif1*$^{-/-}$	0.97	3	
6c(down)	*Shld3*$^{-/-}$	1.0	4	
6c(down)	*53bp1*$^{-/-}$ *Rev7*$^{-/-}$	1.0	3	
6c(down)	*Rif1*$^{-/-}$ *Rev7*$^{-/-}$	1.0	3	
6c(down)	*Shld3*$^{-/-}$ *Rev7*$^{-/-}$	1.0	3	
6c(down)	*Rev1*$^{-/-}$	0.0086	6	
6c(down)	*Rev1*$^{\Delta 9/\Delta 9}$	0.99	5	
6c(down)	*Rev3l*$^{-/-}$	0.15	4	
7b(left)		One-way ANOVA<br>*p* value compared to CH12		F(DFn, DFd)<br>F(6, 20)=19.53
7b(left)	*53bp1*$^{-/-}$	1.0	4	
7b(left)	*Rif1*$^{-/-}$	<0.0001	3	
7b(left)	*Shld3*$^{-/-}$	0.90	4	
7b(left)	*Rev7*$^{-/-}$	0.0003	5	
7b(left)	*Rev1*$^{-/-}$	0.36	3	
7b(left)	*Rev3l*$^{-/-}$	<0.0001	3	
7b(right)		One-way ANOVA<br>*p* value compared to CH12		F(DFn, DFd)<br>F(6, 20)=7.994
7b(right)	*53bp1*$^{-/-}$	0.85	4	
7b(right)	*Rif1*$^{-/-}$	<0.0001	3	
7b(right)	*Shld3*$^{-/-}$	0.090	4	
7b(right)	*Rev7*$^{-/-}$	0.0005	5	
7b(right)	*Rev1*$^{-/-}$	0.26	3	
7b(right)	*Rev3l*$^{-/-}$	0.012	3	
9a		One sample *t*-test<br>*p* value compared to WT		
9a	EV	<0.0001	3	t=44 df=47
9a	Y63A	<0.0001	3	t=21 df=47
9a	W171A	<0.0001	3	t=21 df=47
9a	Y63AW171A	<0.0001	3	t=43 df=47
9a	K129A	<0.0001	3	t=26 df=47
9a	L186A	<0.0001	3	t=7.8 df=47
9a	V85E	<0.0001	3	t=20 df=47
9b(left)		One-way ANOVA<br>*p* value compared to WT		F(DFn, DFd)<br>F(7, 16)=7.201

9b(left)	EV	0.034	3	
9b(left)	Y63A	0.98	3	
9b(left)	W171A	0.94	3	
9b(left)	Y63AW171A	0.043	3	
9b(left)	K129A	0.97	3	
9b(left)	L186A	0.13	3	
9b(left)	V85E	1.0	3	
9b(right)		One-way ANOVA<br>*p* value compared to WT		F(DFn, DFd)<br>F(5, 12)=11.41
9b(right)	EV	0.0007	3	
9b(right)	Y63A	0.13	3	
9b(right)	W171A	<0.0001	3	
9b(right)	Y63AW171A	0.0008	3	
9b(right)	K129A	0.24	3	
9b(right)	L186A	1.0	3	
9b(right)	V85E	0.14	3	
11a		One sample *t*-test<br>*p* value compared to WT		
11a	*53bp1*$^{-/-}$	0.060	3	t=1.9, df=119
11a	*Cd19creRev7*$^{fl/fl}$	<0.0001	6	t=9.5, df=168
11b		One sample *t*-test<br>*p* value compared to WT		
11b	*53bp1*$^{-/-}$	0.15	3	t=1.4, df=169
11b	*Cd19creRev7*$^{fl/fl}$	<0.0001	6	t=18, df=165
11e		Unpaired two-tailed *t*-test<br>*p* value *Cd19cre* compared to *Cd19creRev7*$^{fl/fl}$		
11e	A>G	0.16	5	t=1.5, df=8
11e	A>C	0.35	5	t=0.98, df=8
11e	A>T	0.33	5	t=1.0, df=8
11e	G>A	0.27	5	t=1.2, df=8
11e	G>C	0.29	5	t=1.1, df=8
11e	G>T	0.92	5	t=0.10, df=8
11e	C>A	0.078	5	t=2.0, df=8
11e	C>G	0.33	5	t=1.0, df=8
11e	C>T	0.21	5	t=1.4, df=8
11e	T>A	0.59	5	t=0.57, df=8
11e	T>G	0.65	5	t=0.47, df=8
11e	T>C	0.22	5	t=1.3, df=8
11f		Unpaired two-tailed *t*-test<br>*p* value *Cd19cre* compared to *Cd19creRev7*$^{fl/fl}$		
11f	A>G	0.73	5	t=0.35, df=8
11f	A>C	0.65	5	t=0.47, df=8
11f	A>T	0.07	5	t=2.1, df=8
11f	G>A	0.58	5	t=0.57, df=8
11f	G>C	0.96	5	t=0.057, df=8
11f	G>T	0.87	5	t=0.17, df=8
11f	C>A	0.87	5	t=0.17, df=8

11f	C>G	0.50	5	t=0.70, df=8
11f	C>T	0.59	5	t=0.57, df=8
11f	T>A	0.14	5	t=1.6, df=8
11f	T>G	0.85	5	t=0.19, df=8
11f	T>C	0.77	5	t=0.30, df=8
12b		Unpaired two-tailed *t*-test<br>*p* value compared to Rev7$^{-/-}$		
12b	CH12F3	0.024	8	t=2.5, df=14
12c		Unpaired two-tailed *t*-test<br>*p* value compared to Rev7$^{-/-}$<br>C>T		
12c	CH12F3	0.13	4	t=1,7, df=6
12c		Unpaired two-tailed *t*-test<br>*p* value compared to Rev7$^{-/-}$<br>C>G		
12c	CH12F3	0.12	4	t=1,8, df=6
12c		Unpaired two-tailed *t*-test<br>*p* value compared to Rev7$^{-/-}$<br>C>A		
12c	CH12F3	0.87	4	t=0.17, df=6
13b		Paired two-tailed *t*-test<br>*p* value compared to CH12 EV		
13b	AID	0.39	4	t=1.0, df=3
13b	A3A	0.019	4	t=4.6, df=3
13b		Paired two-tailed *t*-test<br>*p* value compared to *53bp1*$^{-/-}$<br>EV		
13b	AID	0.085	4	t=2.5, df=3
13b	A3A	0.0055	4	t=7.2, df=3
13b		Paired two-tailed *t*-test<br>*p* value compared to *Rev7*$^{-/-}$ EV		
13b	AID	0.012	4	t=5.4, df=3
13b	A3A	0.0011	4	t=12.7, df=3
