# Supplementary material for: REV7 is required for processing AID initiated DNA lesions in activated B cells
Source: Nat Commun. 2020 Jun 4;11:2812. doi: 10.1038/s41467-020-16632-8 (PMC7272641; doi:10.1038/s41467-020-16632-8)
Supplement: Supplementary file 3 — Reporting Summary [file 41467_2020_16632_MOESM3_ESM.pdf]

nature research

Corresponding author(s): Fei-Long Meng

Last updated by author(s): May 5, 2020

Reporting Summary

Nature Research wishes to improve the reproducibility of the work that we publish. This form provides structure for consistency and transparency in reporting. For further information on Nature Research policies, seeAuthors & Referees and theEditorial Policy Checklist .

Statistics

For all statistical analyses, confirm that the following items are present in the figure legend, table legend, main text, or Methods section.

n/a	Confirmed	
☐	☒	The exact sample size (*n*) for each experimental group/condition, given as a discrete number and unit of measurement
☐	☒	A statement on whether measurements were taken from distinct samples or whether the same sample was measured repeatedly
☐	☒	The statistical test(s) used AND whether they are one- or two-sided<br>*Only common tests should be described solely by name; describe more complex techniques in the Methods section.*
☒	☐	A description of all covariates tested
☒	☐	A description of any assumptions or corrections, such as tests of normality and adjustment for multiple comparisons
☐	☒	A full description of the statistical parameters including central tendency (e.g. means) or other basic estimates (e.g. regression coefficient) AND variation (e.g. standard deviation) or associated estimates of uncertainty (e.g. confidence intervals)
☐	☒	For null hypothesis testing, the test statistic (e.g. *F*, *t*, *r*) with confidence intervals, effect sizes, degrees of freedom and *P* value noted<br>*Give P values as exact values whenever suitable.*
☒	☐	For Bayesian analysis, information on the choice of priors and Markov chain Monte Carlo settings
☒	☐	For hierarchical and complex designs, identification of the appropriate level for tests and full reporting of outcomes
☒	☐	Estimates of effect sizes (e.g. Cohen's *d*, Pearson's *r*), indicating how they were calculated

Our web collection on statistics for biologists contains articles on many of the points above.

Software and code

Policy information about availability of computer code

Data collection	Gel Imaging: Tanon 1600; membrane Imaging: Tanon 5200; qPCR:Roche LC96; Microscopy: Olympus BX51; Flow cytometry: Beckman cytoflex; BD FACSAriaII
Data analysis	R Studio v3.5.1; ImageJ v1.52a; Microsoft Excel v16.16.21; GraphPad Prism 7.0a; FlowJo X 10.0.7R2; SSC program 1.0.

For manuscripts utilizing custom algorithms or software that are central to the research but not yet described in published literature, software must be made available to editors/reviewers. We strongly encourage code deposition in a community repository (e.g. GitHub). See the Nature Research guidelines for submitting code & software for further information.

Data

Policy information about availability of data

All manuscripts must include a data availability statement. This statement should provide the following information, where applicable:

- Accession codes, unique identifiers, or web links for publicly available datasets
- A list of figures that have associated raw data
- A description of any restrictions on data availability

HTGTS and SHM sequencing data have been deposited in the NCBI Sequence Read Archive (SRA) with the BioProject accession code: PRJNA590097 (https://www.ncbi.nlm.nih.gov/bioproject/PRJNA590097/). The source data underlying Figs 1a-c, 1e-g, 2a-f, 3a-g, 4a-e, 5a-f, and 6a-b and Supplementary Figs 1a-b, 1d-i, 2c, 3c, 4a-f, 5a, 6b-c, 7b, 9a-b, 11a-f, 12a-c and 13a-b are provided as a Source Data file. All other relevant data are available in the Article, Supplementary Information or from the corresponding author upon reasonable request.

Field-specific reporting

Please select the one below that is the best fit for your research. If you are not sure, read the appropriate sections before making your selection.

☒ Life sciences ☐ Behavioural & social sciences ☐ Ecological, evolutionary & environmental sciences

For a reference copy of the document with all sections, see nature.com/documents/nr-reporting-summary-flat.pdf

Life sciences study design

All studies must disclose on these points even when the disclosure is negative.

Sample size	The sample size has been stated in the figure legends. This was chosen based on previous experience with similar experiments in the literature. We used minimal numbers of animals, which could still allow for generation of statistically meaningful data. No statistical methods were used to predetermine sample size.
Data exclusions	All relevant data were showed. No data were excluded from the analysis.
Replication	All experimental findings in this study have been reproduced at least three times and sometimes even more. In the repeated experiments, similar results were obtained. Use of statistical methods have been described in relevant figure legends. All attempts at replication were successful.
Randomization	For experiments involving mice, no randomization of mice was performed. Mice that were used in all experiments were age and sex-matched where possible. For cell line experiments, samples were grouped based on the genotypes.
Blinding	This study does not require investigators to be blinded to group allocation during data collection and/or analysis. Samples were analyzed with the same protocol by different investigators. The data collection was not subjective.

Reporting for specific materials, systems and methods

We require information from authors about some types of materials, experimental systems and methods used in many studies. Here, indicate whether each material, system or method listed is relevant to your study. If you are not sure if a list item applies to your research, read the appropriate section before selecting a response.

Materials & experimental systems

n/a	Involved in the study	
☐	☒	Antibodies
☐	☒	Eukaryotic cell lines
☒	☐	Palaeontology
☐	☒	Animals and other organisms
☒	☐	Human research participants
☒	☐	Clinical data

Methods

n/a	Involved in the study	
☒	☐	ChIP-seq
☐	☒	Flow cytometry
☒	☐	MRI-based neuroimaging

Antibodies

Antibodies used	Antibodies for western blot:<br>Antibodies for ATM (2873S; Cell Signaling; 1:1000), 53BP1 (NB100-304; NOVUSBIO; 1:1000), RIF1 (ab1213422; Abcam; 1:500), REV7 (A9861; Abclonal; 1:1000), REV1 (sc-393022; Santa Cruz; 1:1000), REV3L (GTX17515; Gene Tex; 1:1000), AID (A16217; Abclonal; 1:1000), MSH2 (ab227941; Abcam; 1:1000), beta-actin (AC028; Abclonal; 1:10000), FLAG (F1804, Sigma; 1:1000),beta-Tubulin (A01030HRP; Abbkine; 1:10000), GAPDH (AB2000; Abways; 1:20000) and Rabbit TrueBlot (18-8816-33; Ebioscience; 1:1000).<br>Antibodies for flow cytometry:<br>PE-conjugated anti-mouse IgA (12-4204-83; Ebioscience; 1:200), APC-conjugated anti-mouse IgM (1020-11S; Southern biotech; 1:200), APC-conjugated anti-mouse B220 (553092; BD; 1:200), FITC-conjugated anti-mouse IgG1 (553443; BD; 1:200), FITC-conjugated anti-mouse IgG3 (553403; BD; 1:200), APC-eFluor780-conjugated anti-mouse B220 (47-0452-82, Invitrogen; 1:200), FITC-conjugated anti-mouse GL7 (144604, BioLegend; 1:200), PE-Cy7-conjugated anti-mouse CD95 (557653, BD; 1:200) and Fluorescein labeled Peanut Agglutinin (FL-1071, Vector Laboratories; 1:500).<br>Antibodies and cytokines for CH12F3 cell lines stimulation:<br>anti-CD40 (16-0402-86; Ebioscience; 1ug/ml); TGF-beta (CA59; Novoprotein; 0.5 ng/ml); IL4 (CK15; Novoprotein; 5 ng/ml)
Validation	For anti-AID antibody (A16217; Abclonal) generated in our lab, the specificity was analyzed by western blot using cytokine activated B-lineage CH12F3 cells and AID deficient CH12F3 cells.<br>For other commercial antibodies, the specificity were also validated by the manufactures as indicated on their web sites.<br>ATM antibody: Western blot analysis of extracts of HeLa, NCCIT and PYS2 cells. 53BP1 antibody: Species Reactivity, Human,

Mouse. Usage: Western blot. RIF1 antibody: Species Reactivity, Mouse. Usage: Western blot. REV7 antibody: Species Reactivity, Human, Mouse. Usage: Western blot. REV1 antibody: Western blot analysis of extracts of HeLa cells. REV3L antibody: Species Reactivity, Mouse. Usage: Western blot. MSH2 antibody: Western blot analysis of extracts of HeLa cells. FLAG antibody: Usage: Western blot. Antibodies for flow cytometry are validated for staining mouse lymphocytes.

Eukaryotic cell lines

Policy information about cell lines

Cell line source(s)	B-lineage CH12F3 cell line was a kind gift from Dr. Tasuku Honjo (Kyoto University), AID deficient CH12F3 cell line is a kind gift from Dr. Kefei Yu (Michigan State University). Other deficient CH12F3 cell lines were deleted with CRISPR/Cas9. HEK293T were from National Infrastructure of Cell Line, SIBCB, with identifier SCSP-502.
Authentication	All cell lines were kept at low passages to maintain their identity. The deletion of genes were confirmed with genomic DNA genotyping-PCR, and RT-PCR/western blot. The overexpression of proteins were confirmed with western blot.
Mycoplasma contamination	All cell lines were tested negative for mycoplasma contamination
Commonly misidentified lines (See ICLAC register)	No commonly misidentified cell lines were used in this study.

Animals and other organisms

Policy information about studies involving animals; ARRIVE guidelines recommended for reporting animal research

Laboratory animals	For the study we used Aicda-/- mouse line, which was a kind gift from Dr. Tasuku Honjo (Kyoto U), to purify splenic primary B cells. RBRC No.: RBRC00897; Strain name: B6.Cg-Aicda<tm1Hon> (N10)/HonRbrc.<br>53bp1-/- and Atm-/- mouse lines were kind gifts from Dr. Frederick W. Alt (Harvard Medical School). Cd19cre mouse line was a kind gift from Dr. Klause Rajewsky (Max Delbrück Center)<br>Rev7 floxed mouse line was constructed by this study.<br>6-8 week old mice were used. Both male and female mice were used for the study.
Wild animals	The study did not involve wild animals.
Field-collected samples	The study did not involve samples collected from the field.
Ethics oversight	All animal experiments were performed under protocols approved by the Institutional Animal Care and Use Committee of Shanghai Institute of Biochemistry and Cell Biology.

Note that full information on the approval of the study protocol must also be provided in the manuscript.

Flow Cytometry

Plots

Confirm that:

- [x] The axis labels state the marker and fluorochrome used (e.g. CD4-FITC).
- [x] The axis scales are clearly visible. Include numbers along axes only for bottom left plot of group (a 'group' is an analysis of identical markers).
- [x] All plots are contour plots with outliers or pseudocolor plots.
- [x] A numerical value for number of cells or percentage (with statistics) is provided.

Methodology

Sample preparation	For flow cytometry assay, cells were collected and washed with pre-cooling FACS buffer (1xPBS buffer with 2.5% FBS (v/v)). Then resuspended cells with diluted fluorescence-conjugated antibodies in FACS buffer and incubated at room temperature for 10 minutes. Washed the cells with FACS buffer and resuspended in 100-200 microliter FACS buffer for flow cytometry.
Instrument	Samples were acquired on a CytoFLEX Flow Cytometer (Beckman Coulter); Samples were sorted on a BD FACSAriaII (Becton Dickinson)
Software	CytoFLEX was used to collect the flow cytometry data. FlowJo X 10.0.7R2 was used to analyze the flow cytometry data.
Cell population abundance	Due to the low abundance of the relevant cell populations, the abundance of the populations in the post-sort fraction was not assessed.
Gating strategy	Using the FSC/SSC plot, a gate was drawn to select activated primary B cells or B-lineage CH12F3 cells and to exclude debris.<br>Cell cycle analysis: fixed cells were gated as singlets (FSC-A vs Hoechst-W) and assessed for DNA content by staining Hoechst and EdU.<br>Cell apoptosis analysis: all cells were gated and assessed by staining PI and AnnexinV-FITC, we gated on AnnexinV positive and PI

negtive cells as apoptotic cells.
For activated primary B cells, using the APC/FITC plot, we gated on B220 positive cells and IgG1 positive cells.
For B-lineage CH12F3 cells, using the APC/PE plot, we gated on IgM positive cells and IgA positive cells.
For Germinal center B cells,using the APC-eFluor780 plot, we gated on B220 positive cells, while B220 positive cells were subgated using the FITC/PECy7 plot, we gated on GL7 positive and CD95 positive cells as Germinal center B cells.
Positive and negative populations for gating were defined using empty vector control treated samples.

☒ Tick this box to confirm that a figure exemplifying the gating strategy is provided in the Supplementary Information.
